# DrosoPHILA: A Partnership between Scientists and Teachers That Begins in the Lab and Continues into City Schools

**DOI:** 10.1523/ENEURO.0263-22.2022

**Published:** 2023-02-13

**Authors:** Kaitlin M. Laws, Ent Natale, Edward A. Waddell, Jamie R. Shuda, Greg J. Bashaw

**Affiliations:** 1 Department of Neuroscience, Perelman School of Medicine, University of Pennsylvania, Philadlephia, PA 19104; 2 Institute for Regenerative Medicine, Perelman School of Medicine, University of Pennsylvania, Philadelphia, PA 19104

**Keywords:** *Drosophila*, neurodevelopment, outreach

## Abstract

Here, we describe the development, structure, and effectiveness of an outreach program, DrosoPHILA, that leverages the tools of our fly neurodevelopmental research program at the University of Pennsylvania to reinforce the biology curriculum in local public schools. DrosoPHILA was developed and is sustained by a continued collaboration between members of the Bashaw lab, experienced outreach educators, and teachers in the School District of Philadelphia. Since the program’s inception, we have collaborated with 18 teachers and over 2400 students. Student outcome data indicates significant positive attitude shifts around science identity and grade-appropriate knowledge gains.

## Significance Statement

Outreach programming creates connections between scientists and their communities while expanding students’ perception of what science entails and who practices it. As such, outreach programming can act as one part of a multipronged approach to diversify the scientific workforce. To build sustainable, effective science outreach curricula, scientists should seek input from both teachers in their communities and experienced outreach educators. Here, we present our outreach program, DrosoPHILA, as a model for such partnerships. By explaining the program’s development and making our supporting materials available, we hope to facilitate the creation of similar programs across different subject areas.

## Introduction

Advances in emerging scientific fields have revolutionized STEM disciplines, creating new science-based industries and careers. In the United States, however, the demographic profile of the scientific workforce does not reflect that of the country (Segarra et al., 2020; Tilghman et al., 2021; Unguez et al., 2022). This lack of representation creates barriers to entry to potentially lucrative careers and demonstrably affects the scope and objectivity of scientific inquiry (Tilghman et al., 2021). Improving diversity in STEM will require action in many policy arenas and at every level of scientific training. Nevertheless, a career in science must begin with an interest in science. While exposure to inquiry-based laboratory activities often awakens student interest in science, many public schools in the United States lack the resources to offer such experiences. This issue is compounded in city schools, which often serve students from low-income and historically marginalized communities who are underrepresented in the scientific workforce. For example, the School District of Philadelphia, the public school district that serves the city in which our university is located, is a 100% Title One District that provides free or reduced-price meals, a low-income indicator, to all students. In the 2021–2022 school year, 74% of students identified as African American or Hispanic (PSSA & Keystone Performance, 2022). Without a full understanding of scientific career options, only ∼20% of students pursuing bachelor’s degrees in the United States typically declare a STEM major (Sharkawy, 2015; Riegle-Crumb et al., 2019). Students from underrepresented groups are less likely to pursue STEM careers when they are educated in under-resourced schools with inadequate STEM preparation (Zhang and Barnett, 2015; Rotermund and Burke, 2021). This educational inequity exacerbates the existing nationwide underrepresentation in science.

We are seeing this play out in Philadelphia city schools. Many urban students traditionally score below grade level on standardized tests in science: in the 2018/19 school year, for example, 72.4% of Black students scored below grade level expectations on the state science assessment (PSSA & Keystone Performance, 2022). The biology curriculum is particularly challenging because the vocabulary is new, the content is dense, and students struggle to relate the information to their own background knowledge. The disconnect from what students learn to its application to their daily life only widens the knowledge gap (Bouillion and Gomez, 2001). As scientists and educators, we understand that science, rather than a set of facts, is a practice. Thus, student learning itself must also be an active process that connects to problems and situations relevant to students’ lives (Chaplin and Manske, 2005). A hands-on approach to the science curriculum may not only teach valuable skills that could generalize to new subjects and situations but can also spark greater student interest and motivation in science coursework (Faris, 2008; Foley and McPhee, 2008; Hurney, 2012; Swarat et al., 2012).

Members of the scientific community, including faculty and trainees at local colleges and universities, are well-positioned to promote experiential learning at area public schools. Outreach sponsored by local colleges and universities can provide public school students with first-hand knowledge of the scientific process, reinforce learning objectives, improve science identity, and connect them to scientists in their community. We are a team of experienced outreach educators (J.R.S., E.N.) and scientists (K.M.L., E.A.W., G.J.B.) who sought to leverage the resources of the University of Pennsylvania to support Philadelphia students in becoming scientists in their own classroom.

The Bashaw lab uses *Drosophila* and mice as model organisms to study the genetics of neural circuit development. Our project targets students enrolled in high school biology classes as this is where *Drosophila* neurogenetic experiments align with state and local academic science standards. Many of the feature of flies that have made them popular research organisms (their small size, low maintenance cost, and the wide range of biological problems that they can be used to address) also make them well-suited for high school science classrooms. Indeed, several other groups, including eClose in Philadelphia (https://ecloseinstitute.org/) and the Manchester Fly Facility in the United Kingdom (droso4schools.wordpress.com) run successful outreach programming with flies (Patel and Prokop, 2017; Patel et al., 2017; Waddell et al., 2021; Hanley et al., 2022).

DrosoPHILA is our two-part high school curriculum built by teachers in the School District of Philadelphia, our local public school district, with input and support from members of our lab and experienced outreach educators. The modules in our outreach program, Flies on Ice and Roundabout We Go!, highlight the utility of *Drosophila* as a model organism for neurobiology research while reinforcing students’ understanding of the scientific method and quantitative reasoning. Further, they create an opportunity for students to engage with science as it is practiced and for students to see themselves as scientists. By bringing teachers into the lab and scientists into the classroom, our program fosters mutual insight and understanding of the unique perspectives of students, teachers, and scientists.

Here, we describe our curriculum and key features of its development with an eye toward helping scientists interested in building similar programs related to their own research. In Philadelphia, high school biology students are expected to learn basic biology principles: the chemical basis of life, genetics, evolution, and the scientific process. DrosoPHILA embeds this material in an exploratory, real-life experience that reinforces students’ understanding of the science content required at no cost to students or city schools. By partnering in our own research lab and in the classroom, we are reaching both teachers and their students.

## Materials and Methods

### Module lesson plans

Complete lesson plans and DrosoPHILA student workbooks are available (Extended Data 1, 2, 3, 4). For assistance in replication, please contact J.R.S. (jamie.shuda@pennmedicine.upenn.edu). Formalized collaboration will provide additional support to your effort, including identification of teacher collaborators, the establishment of evaluation plans for your program, and help training interested university partners.

10.1523/ENEURO.0263-22.2022.ed1 Extended Data 1 Lesson plans for Flies on Ice. Download Extended Data 1, ZIP file.

10.1523/ENEURO.0263-22.2022.ed2 Extended Data 2 Flies on Ice student workbook. Download Extended Data 2, ZIP file.

10.1523/ENEURO.0263-22.2022.ed3 Extended Data 3 Lesson plans for Roundabout We Go! Download Extended Data 3, ZIP file.

10.1523/ENEURO.0263-22.2022.ed4 Extended Data 4 Roundabout We Go! student workbook. Download Extended Data 4, ZIP file.

10.1523/ENEURO.0263-22.2022.t3-1 Extended Data Table 3-1 A list of all premodule and postmodule survey questions used to analyze module effectiveness. Download Table 3-1, DOCX file.

### Evaluating module effectiveness

We used a Google form to anonymously survey students before and after each module. Our surveys contained both multiple-choice questions to test student knowledge and Likert scale questions to assess students’ attitudes toward science. Our question design was informed by previously published outreach survey questions (Shuda et al., 2016). For knowledge questions, we compared the percentage of students who answered a question correctly before the module to the percentage who answered it correctly after the module, evaluating statistical significance in GraphPad Prism by χ^2^ analysis. For Likert scale attitude questions, we compared the distribution of Likert scores before and after the modules in Microsoft Excel by Student’s *t* test with a *post hoc* Bonferroni correction for multiple comparisons. We generated graphs in GraphPad Prism. Importantly, our relationship with the School District of Philadelphia precludes the inclusion of a control group; our goal is to provide a hands-on experience to as many students as possible.

### Approach: a two-phase model for outreach development

Where should scientists begin when considering how to adapt their research to high school outreach programming? While there are many potential strategies, working relationships with school district teachers has been critical for the implementation and sustainability of our program. Support from the National Science Foundation allowed us to offer stipends for teachers to spend time in our lab, removing potential financial barriers to teacher participation. This project is built off the proven principles of university-school collaborations used in Project BioEYES (https://www.bioeyes.org/), an award-winning K-12 outreach program established in 2002 by Dr. Jamie Shuda and Dr. Steven Farber. BioEYES has served over 155,000 students to date and currently operates in 16 sites, including several internationally. The principles that have led to BioEYES’ sustainability that have been adopted by our team include building relationships and the science competence of collaborating teachers (Shuda et al., 2019), as well as developing sustainable personnel, funding, and evaluation strategies at the university (Shuda et al., 2016). Including this outreach project as the “broader impacts” component of an NSF grant proposal secured the funding to develop the first two phases of DrosoPHILA, described below.

#### Phase 1

Beginning in 2014, teachers from the School District of Philadelphia conducted paid summer research in the Bashaw lab while helping to develop outreach modules (Box 1). We recruited participants through the network of teachers in the school district who had previously taken part in the Project BioEYES program. Past participation in BioEYES gave teachers an idea of what to expect of an outreach experience from our group. While in our lab, teachers contributed to an ongoing genetic screen, working side-by-side with laboratory trainees to understand the genes involved in circuit development in the *Drosophila* nervous system. By bringing teachers into our laboratory, we were able to draw on their expertise and familiarity with Next Generation Science Standards as well as our state-specific biology standards. Teachers have an unparalleled understanding of the subject areas where student learning could be supported with hands-on experiments. Further, as other outreach groups have noted, long-term relationships between scientists and teachers can lead to a beneficial professional exchange in which teachers are exposed to contemporary scientific contexts and scientists hone their communication skills (Patel et al., 2017).

Box 1 Teachers participating in DrosoPHILA comment on their involvement.

Teacher 1 (2018)

“The longer a teacher is in the classroom, the more they become expert at communicating science to students and engaging them in science practices, but it comes at a cost of losing touch with contemporary science knowledge and skills. It’s incredibly important and impactful to give science teachers opportunities to be in active research labs. It takes a summer of daily contact with graduate students to understand how they think about their work, solve problems, and use technology. The Bashaw lab was a place to experience the very important implications (for early brain development) of some foundational topics like genetics and signaling pathways. And it was rewarding to work on translating the lab’s work into experiences for high school students that additionally allow them to interact directly with current researchers that are a lot more like them than a long-gone Austrian monk.”

Teacher 2 (2016)

“I spent a summer in the…lab and learned so many practical skills that I was able to set up my own high school Drosophila research lab where my students can design and conduct their own research experiments. My experience in the Bashaw lab has enabled me to teach research skills to my high school students at a much more sophisticated level and prepare them more fully for a career in the STEM field.”

Teacher 3 (2015, 2018)

“As a veteran teacher, I have found that most professional development focuses on classroom management and how to differentiate lessons. While important, pedagogy is only half of teaching. The other half is content knowledge. I wanted to broaden my understanding of science and learn more about how professionals in the field discover new things in the world. With DrosoPHILA I got the opportunity to do just that. I worked alongside researchers from the…lab, as well as other district teachers, to develop curriculum that used concrete practices to bring abstract science concepts to life.

DrosoPHILA generates positive outcomes. Students get excited about the experiments and are more engaged to learn about exocytosis when it is related to the flies’ inactivity on ice. They ask probing questions and connect the phenomena to experiences in their own lives. I look forward to the future, growth and implementation of DrosoPHILA throughout the School District of Philadelphia.”

#### Phase 2

As we developed modules for our outreach program with teachers, we then piloted, refined, and disseminated these lessons to city schools. At the onset of the classroom dissemination, volunteers from the Bashaw lab routinely led classroom visits for teachers who had participated in research in our lab, often accompanied by other graduate students and postdoctoral fellows as well as local outreach affiliates. To expand our program to classrooms outside of this initial cohort, we hosted a 2-d professional development program during the summer of 2018. During the event, six teachers from five different district schools visited our lab and had the opportunity to participate in each of our modules. Teachers received Act 48 credit for their participation, which allows teachers to fulfill state-specific professional development requirements through their attendance. At the end of the session, we scheduled teachers for classroom visits for the 2018–2019 school year. An expansion of our reach required additional personnel, and in the fall of 2019, with the support of the NSF and matching contributions from the University of Pennsylvania Perelman School of Medicine Department of Neuroscience, we hired a designated outreach educator (E.N.), who took over organizing and leading classroom visits. At the onset of the COVID-19 pandemic, we transitioned our program online. Though we reached nearly 500 students by remote instruction, we felt that we lost the excitement and other benefits of classroom visits and hands-on experimentation, including possible benefits to outreach volunteers themselves. In the Fall of 2021, we resumed classroom visits, reaching >800 students across 21 classrooms in the 2021–2022 school year.

#### Phase 3

There is a potential for phase three of this project. This would entail opening the Bashaw lab summer research opportunity to new teachers so that they can learn about current research, refine the DrosoPHILA curricula, and develop additional modules for the program. To increase the reach of our program, we could also “graduate” teachers who have had Flies on Ice in their classroom for two or more years to “model teacher” status. Model teachers would continue to receive materials for the module from us but implement the lesson without classroom support from an outreach educator. Although they may still get assistance from program volunteers, the teacher will take ownership of delivering the content. The model teacher program allows the outreach educator to support new classrooms during the same week, expanding our reach despite a limited budget and a set number of school weeks per year. We are currently piloting this approach to Flies on Ice with three teachers. This has been the primary growth mechanism for Project BioEYES over the past 20 years (Shuda et al., 2016).

#### Modules: two experiments that use *Drosophila* to reinforce student learning

##### Module 1: Flies on Ice

**Figure 1.**
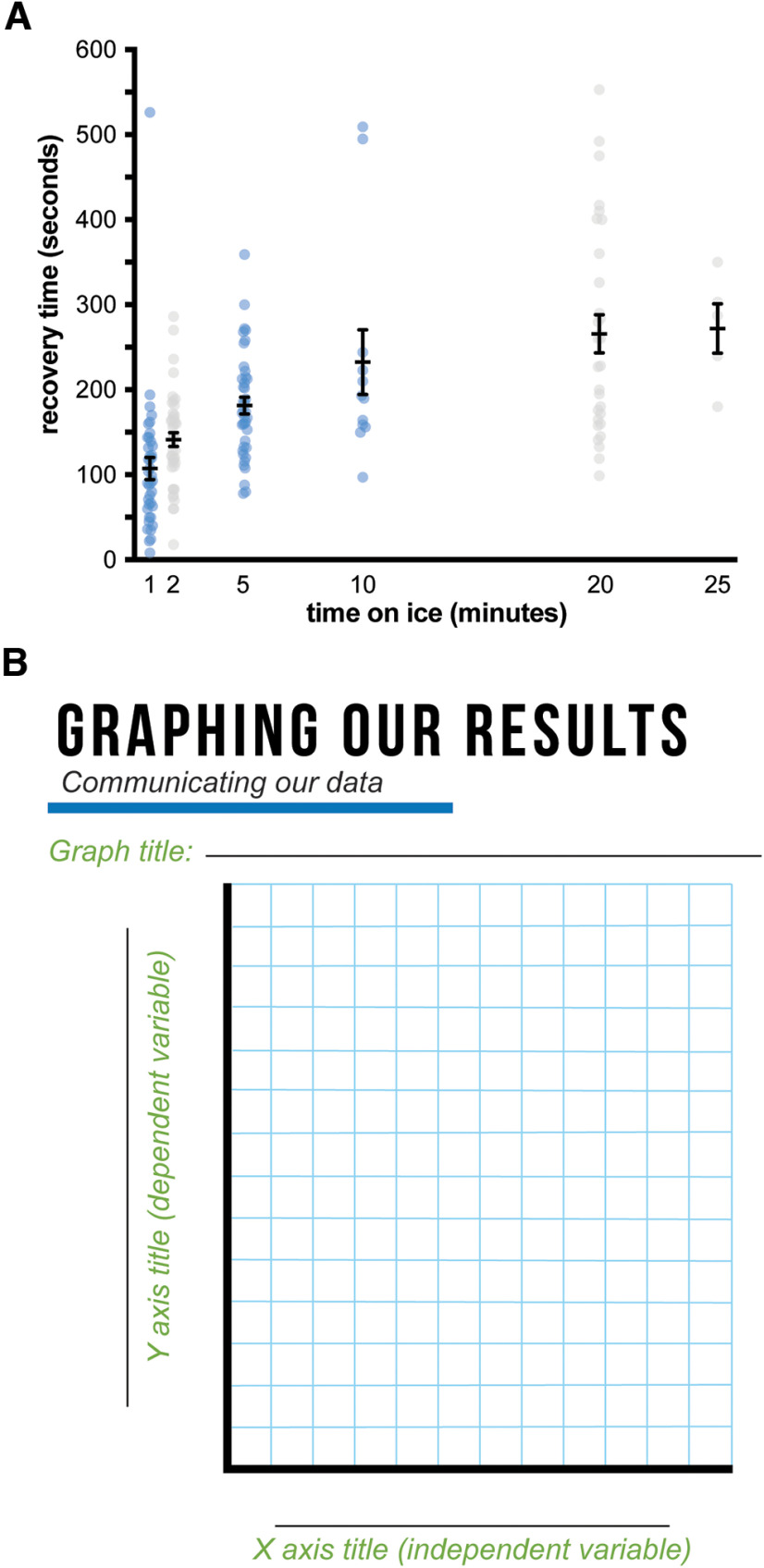
Graphing data collected during the Flies on Ice module. ***A***, Relationship between the amount of time flies spend on ice and their recovery time as documented in School District of Philadelphia classrooms in 2019. Each point represents a single measurement taken from a student group. Each blue point represents a time point for which students collect data in the current version of Flies on Ice; those in gray show other data collected in the past. Error bars represent SEM. *N* = 40, 43, 40, 14, 12, 30, 5. Lesson plans for Flies on Ice are presented in Extended Data 1. ***B***, A sample page from the Flies on Ice workbook (Extended Data 2) in which students graph their group data, then compare it to classroom and program-wide data like that described above.

At the beginning of their biology coursework, students learn about the importance of the scientific method. In our first module of the school year, we provide students with an opportunity to apply the scientific method to a hands-on experiment that we call “Flies on Ice.” Flies on Ice also introduces students to *Drosophila* as a model organism that scientists use to study nervous system development. Over the course of 3 d, students build the skills necessary to address our scientific question: how does the time flies spend anesthetized on ice affect their recovery time? On day 1, we introduce students to the concept of model organisms, and they learn why scientists have been using *Drosophila* as a model for over a century. Next, they receive a virtual tour of the Bashaw lab and see how practicing scientists use fruit flies to understand how the nervous system develops. Students handle vials of fruit flies and make observations about their behavior at different stages of development, learning that the nervous system is responsible for these behaviors. Finally, we lead students in a pilot experiment to help them develop a hypothesis as to how fly behavior will change when we put them “on ice.” In this experiment, flies are transferred from vials containing food to empty vials. These vials are then submerged in cups filled with ice for 1 min. Upon removing the flies from the ice cup, students make observations about how fly behavior changes and discuss whether this evidence supports their initial hypotheses. On days 2 and 3, we direct students to change their scientific question, instead asking them to predict how the amount of time flies spend on ice will affect recovery time from the ice exposure. During experimentation, volunteers circle the room to discuss common observations, including that the flies appear to be dead. The experiment culminates with students graphing the average time it takes for flies to recover from different times spent on ice, then comparing their data to that collected (1) by the class and (2) over the course of our program (Fig. 1). Students observe that while the relationship between the amount of time flies spend on ice and recovery time starts out as linear, recovery time plateaus at later time points. Together, these observations provide a jumping-off point for a discussion of the scientific method and the importance of replication and specific definitions for experimental parameters. Our team also leads a conversation about how students may have reached different conclusions (e.g., bias, differences in methodology) that reinforce their classroom instruction on the scientific method.

##### Module 2: Roundabout We Go!

**Figure 2.**
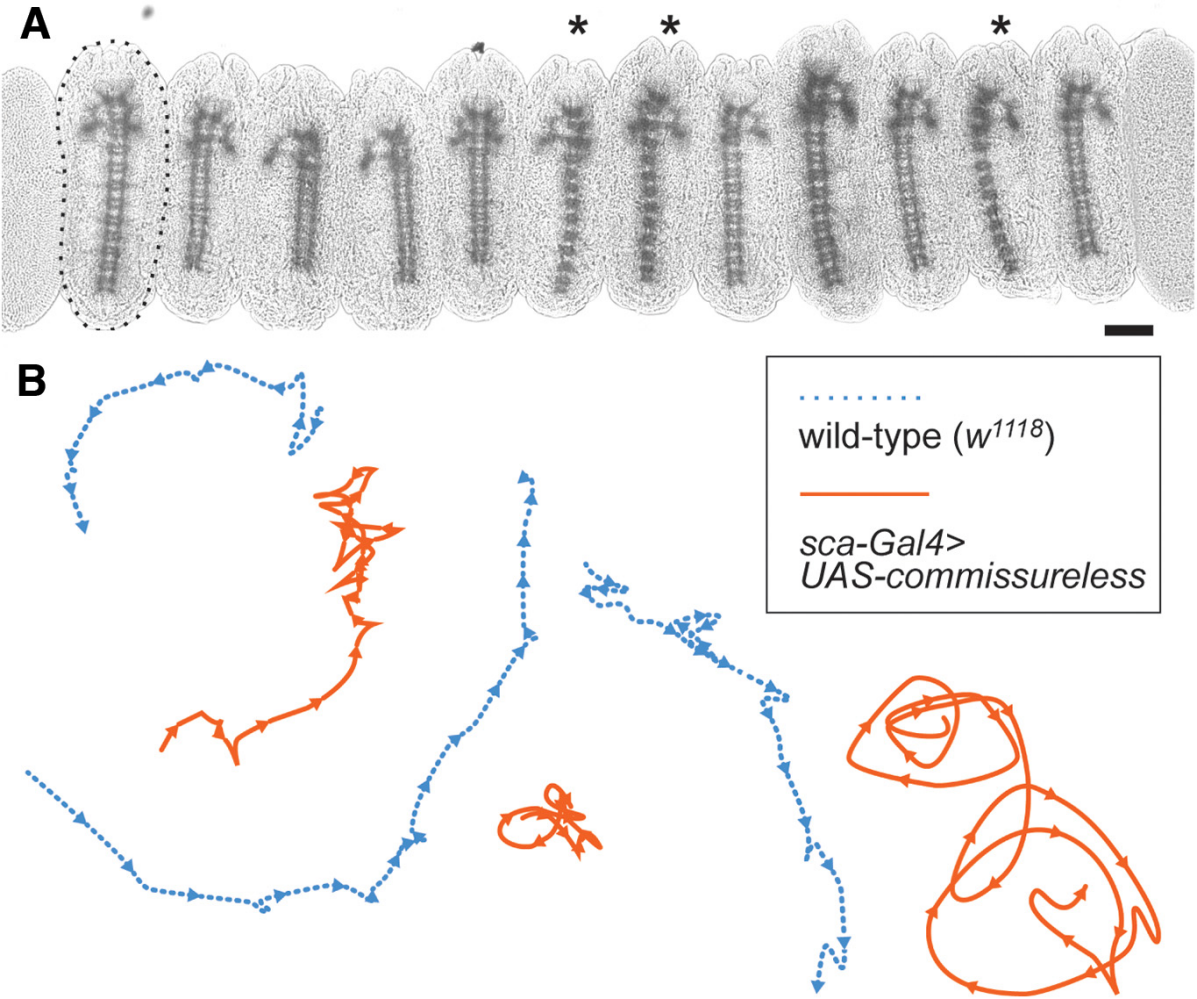
Roundabout We Go!: linking nervous system morphology to behavior. ***A***, Micrograph of 12 whole-mounted *Drosophila* embryos collected from a *roundabout1/CyO* x *roundabout1/CyO* cross. Embryos were stained to visualize their ventral nerve cords under bright field microscopy. One embryo is outlined with a dashed line. Asterisks indicate embryos with the *roundabout1* mutant phenotype; remaining embryos have the wild-type phenotype. Anterior is up. Scale bar: 100 μm. ***B***, Illustration of observed crawling patterns of wild-type larvae (*w^1118^*; dashed blue line) and larvae lacking functional neuronal Roundabout1 (*sca-Gal4>UAS-commissureless*; solid orange line). Students apply diluted food dye to larvae, blinded to genotype, to compare the crawling patterns of these larvae on thermal paper. Then, students use their knowledge of nerve cord morphology to predict which larvae are mutant. Lesson plans for Roundabout We Go! are presented in Extended Data 3, and the Roundabout We Go! workbook is presented in Extended Data 4.

The biology curriculum in the School District of Philadelphia includes a section on transmission genetics, a subject to which we owe significant scientific debt to *Drosophila* research. Hands-on experimentation with flies is therefore a natural fit for reinforcing concepts introduced in this section of the curriculum. In Roundabout We Go!, students investigate the link between genes and behavior through a series of observations and experiments. On day 1, students watch videos of humans and mice with uncoordinated behavior and learn that they both have pathogenic variants in genes that control nervous system development. We discuss how gene expression guides the formation of neural circuits, which is the major focus of research in the Bashaw laboratory, and how model organisms can be used to investigate the genetics of circuit development. On day 2, students use microscopes to observe whole-mount slides of *Drosophila* embryos selected from crosses of parents heterozygous for recessive mutant alleles of genes that control nervous system development (namely, *roundabout*, *slit*, and *commissureless*, three genes studied extensively in the Bashaw lab; Fig. 2*A*). We select embryos to illustrate Mendelian ratios (i.e., one quarter of embryos on each slide have a mutant phenotype). Thus, in addition to understanding how flies can be used to study changes in nervous system development, this exercise is designed to reinforce student understanding of transmission genetics. On day 3, we ask students to consider how structural changes to the nervous system such as those they observed in *roundabout* mutant embryos may affect larval crawling. Students test their hypothesis, blinded to larval genotype, using food coloring to trace larval crawling patterns on thermal paper (Fig. 2*B*). We then ask students to consider how they might quantify their observations, offering up examples from primary literature (Berni, 2015; Kinold et al., 2018).

##### Module scaffolding and delivery

Each module is scaffolded by grade-appropriate presentations, student-driven experimentation, and guided data analysis. Our team, which consists of an outreach educator and volunteer scientists, leads at least two 50-min class periods of instruction, which are complemented by PowerPoint presentations. We bring all the materials necessary to conduct experiments to the school, but draw on school resources when possible (e.g., when available, we use school-owned microscopes). Students receive workbooks to allow them to actively engage with the presentation and to record and reflect on their data (Extended Data 2, 4).

We ask that teachers who participate in our program schedule Flies on Ice early in the school year, when students are reviewing the scientific method, and Roundabout We Go! for the second half of the school year, after students have studied genetics. Restricting the timeframe in which we offer each module situates our lessons appropriately in the science curriculum and simplifies preparation and module delivery for our team. In between DrosoPHILA modules, the teachers are scheduled to complete the weeklong BioEYES’ program. During BioEYES, students explore Mendelian genetics by mating pairs of phenotypically different zebrafish (albino and wild-type) and raising the embryos to identify if their offspring will have pigment. Students are introduced to the complexity of inheritance and how changes in gene expression can lead to observable phenotypes. This sequence of interventions is mutually reinforcing: all depend on student observations of model organism biology, and the zebrafish developmental genetics presented in BioEYES provides a strong foundation for the genetics in Roundabout We Go! Although they were developed with Pennsylvania-specific academic standards in mind, the teacher-informed design of our modules, along with the broad use of *Drosophila* as a model organism, makes them suitable for school districts across the United States, as well as schools in other countries.

## Results

**Figure 3.**
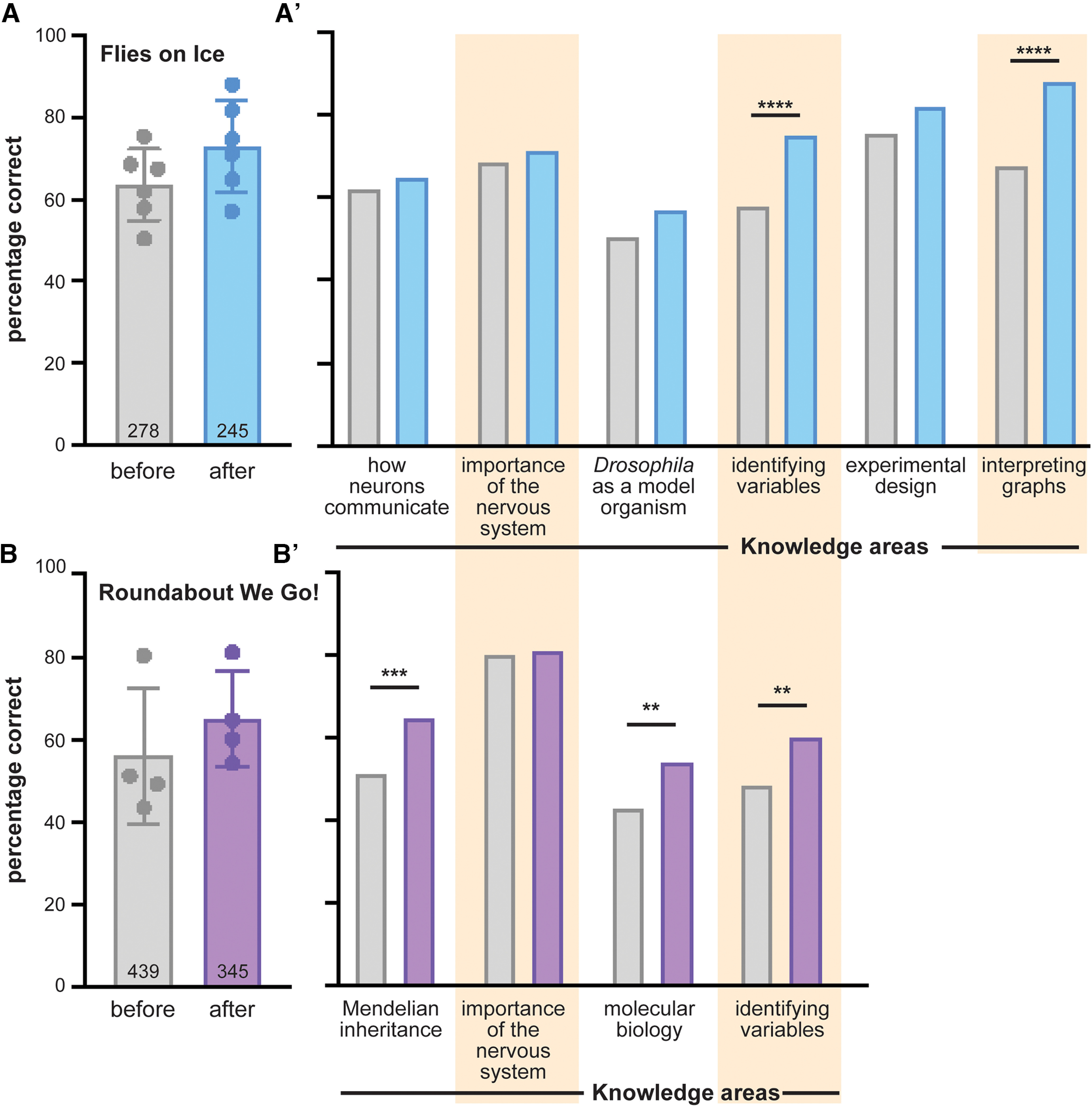
Improved science proficiency after DrosoPHILA participation. ***A***, Percentage of knowledge-based survey questions answered correctly before (gray) and after (blue) Flies on Ice. Each point represents an average from a different question, and error bars represent SD. The sample size reported inside the bar is the minimum number of students who answered the survey question. The survey questions are presented in Extended Data Table 3-1. ***A’***, Percentage of students who answered each knowledge question correctly before (gray) and after (blue) participating in Flies on Ice. ****p < 0.0001, χ^2^ analysis. ***B***, Percentage of knowledge-based survey questions answered correctly before (gray) and after (purple) Roundabout We Go! Each point represents an average from a different question, and error bars represent SD. The sample size reported inside the bar is the number of students who answered the survey question. ***B’***, Percentage of students who answered each knowledge question correctly before (gray) and after (purple) participating in Roundabout We Go! **p < 0.01, ***p < 0.001, χ^2^ analysis.

Our program seeks to improve student science proficiency and shift student perceptions of who practices science and what that practice entails. To determine whether we achieved these goals, we surveyed students before and after module delivery using a Google form (Extended Data Table 3-1). After receiving in-person instruction from our outreach team, students had increased proficiency in several topic areas targeted by our modules (Fig. 3). For example, after participating in Flies on Ice, students were more likely to correctly identify experimental variables and interpret graphs than they were before the module (Fig. 3*A*,*A’*). These concepts are regularly tested on state science examinations, and DrosoPHILA reinforces student understanding by allowing them to apply their knowledge to a real-life phenomenon. Similarly, following Roundabout We Go!, students demonstrated increased proficiency in Mendelian inheritance and molecular biology, two topics that are central to that module (Fig. 1*B*,*B’*). We note that in each module, several survey questions indicate a high level of understanding preintervention, suggesting that students already had strong foundations in these topics. This may explain why we do not observe knowledge gains in some topic areas, including the importance of the nervous system and how neurons communicate with each other. Indeed, because DrosoPHILA modules are aligned with the high school biology curriculum, a high starting level of subject-matter knowledge might be expected; teachers may prepare for our experiment-based visits by reviewing subject matter with their students. Regardless of the reason, the high starting subject-matter knowledge provides us with important context as we seek to improve on our existing program and develop new modules. Future studies could address knowledge gains over the course of both modules.

**Figure 4.**
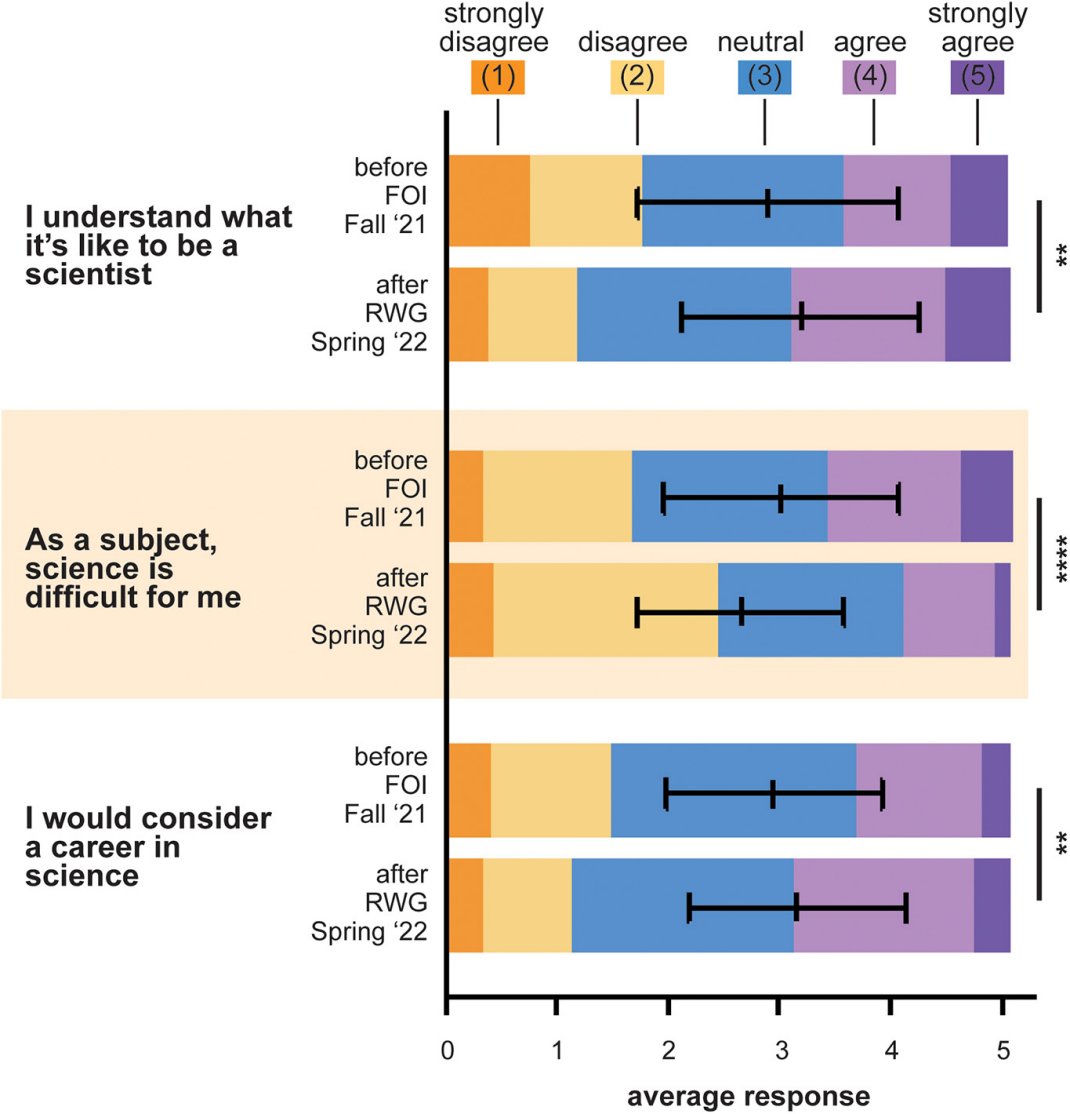
Positive shifts in science identity over the course of a school year with our outreach program. Students were asked the same Likert scale questions regarding their attitude toward science before participating in Flies on Ice (FOI, Fall 2021) and after they completed Roundabout We Go! (RWG, Spring 2022). Lines represent the mean responses, and error bars represent SD. Bins reflect the proportion of students who responded with each number. In Fall 2021, 281 students responded to each question. In Spring 2022, 345 students responded to each question. **p < 0.01, ****p < 0.0001, Student’s *t* test with Bonferroni correction for multiple comparisons.

An equally important goal of our program is to increase our students’ science identity. By connecting students to scientists in their community to perform hands-on experiments, we hope to expand their perception of who can and does practice science. We surveyed students regarding their perception of science before and after each module, ultimately capturing changes in attitudes over the course of the school year (Fig. 4). Before participating in our program, students were most likely to respond neutrally to the statements “I understand what it’s like to be a scientist” and “I would consider a career in science.” After our second module, a higher proportion of students indicated that they agreed with these statements, suggesting improved science identity. Similarly, students were less likely to describe science as a difficult subject for them after participating in our outreach modules. Because this analysis involves long-term assessment of student attitudes, we cannot exclude the possibility that the teachers who participate in our program are particularly attuned to the issue of science identity and prioritize it in their independent activities. Nevertheless, this analysis suggests that involvement with our program correlates with increased science identity, an observation shared by Project BioEYES (Shuda et al., 2016, 2019).

## Discussion

Systemic inequities in education, exacerbated by sociopolitical and historic factors, have led the United States to accumulate significant educational debt toward minority students (Ladson-Billings, 2006). This debt is particularly pronounced in urban settings like Philadelphia, where education spending per student is significantly less than in neighboring suburbs (Ladson-Billings, 2006). Outreach activities are one avenue by which well-resourced colleges and universities like our own may work to repay this educational debt in their communities. STEM outreach activities should both reinforce the learning goals of the classroom and expand students’ understanding of what it means to be a scientist and who becomes scientists. While many scientists, especially at the trainee level, are eager to participate in outreach, building successful and sustainable programming requires input from many stakeholders and significant financial support. By involving teachers at the ground level of our program, we hoped to develop a system in which teachers are empowered to teach independently rather than depend on one-off visits from scientists. This structure would extend the reach of our program past our own personnel limitations. In addition to the actions of teachers, experienced outreach educators, and research scientists, our program is sustained by funding from the NSF and our lab’s home department. This funding allows us to have a designated outreach educator who brings additional perspective and experience to the team. To sustain our program at its current level, we will seek to obtain funds through research and education grants and continue to meet the curriculum needs in city schools. One challenge to the long-term viability of outreach programming is the high turnover rate of the scientific workforce in academia, which consists primarily of short-term trainees. Therefore, having an employee who specifically manages and teaches this program is especially important. To grow and sustain our program, expanding our model teacher program, recruiting new educators to collaborate, and maintaining a university outreach educator would be necessary.

Our reflections on our outreach program have focused on its effectiveness in improving science proficiency and identity in high school students. Several studies, however, have demonstrated a link between undergraduate student participation in outreach activities, science identity, and persistence in science (Gubbels and Vitiello, 2018; Saravanapandian et al., 2019). While little is known regarding the role of outreach participation in graduate and postdoctoral training, scientists at all career stages may relish the opportunity to connect with the wonder of younger students and hone their science communication skills. Indeed, the structure of the workforce in academic science, where research is conducted by trainees with a wide range of career goals, makes us particularly well-equipped to serve in an outreach capacity: we have firsthand knowledge of the variety of careers in science. Furthermore, trainees who participate in outreach are empowered to speak as experts in the field, an experience that may be particularly potent for scientists from minoritized groups and/or those struggling with imposter syndrome. We suspect that recognition of outreach activities as important manifestations of science identity could reaffirm a culture of community in the laboratory. In the future, we plan to expand our analysis of our program to encompass the effect of outreach participation on volunteers themselves.

## Synthesis

Reviewing Editor: Lynda Erskine, University of Aberdeen Decisions are customarily a result of the Reviewing Editor and the peer reviewers coming together and discussing their recommendations until a consensus is reached. When revisions are invited, a fact-based synthesis statement explaining their decision and outlining what is needed to prepare a revision will be listed below. The following reviewer(s) agreed to reveal their identity: Andreas Prokop, Megan Davey.

Thank you for submitting your manuscript describing your outreach initiatives. Both reviewers are agreed that the manuscript nicely encompasses central tenets of high quality science outreach and public engagement with research. However, they both have suggestions for improving the impact of the manuscript and for enabling the adoption of such approaches by others. In particular they would like more detail on the links to your research and why the outreach was undertaken, as well as more understanding of the target audience and how this may influence the outcomes. Detailed comments from both reviewers are listed below.

Reviewer 1:

I applaud the authors for their impressive school outreach initiative and would love to see this work published. However, substantial improvements are necessary before publication should be granted. Too little information is provided to judge and understand the work, and the way the article is organised does not help its case. Substantial rewriting would be required, but I hope the authors are willing to make that extra effort to give their work the adequate recognition. Happy to discuss this with the authors: Andreas.Prokop@manchester.ac.uk.

Detailed comments:

l.3 It is never made clear how the activities link to the authors’ own research.

The introduction is far too short and lacks important information, as clearly illustrated by the fact that there are no references at all. Why is there a need for science education. Given the curriculum, what are the short-comings and how can scientists help? What aspects of the curriculum are being targeted and why? Why does Drosophila provide a good means to fulfil this task? What other initiatives are already out there, and how does this initiative complement existing resources and strategies. Please, find comprehensive information on existing resources here: https://droso4public.wordpress.com/resources/#Teaching. I also recommend our article as an example of strategy development (which is mentioned at a later stage: http://doi.org/10.1016/j.semcdb.2017.07.025 ) but would offer opportunities to compare/contrast the different approaches: sending scientists as teaching assistants into schools versus having teachers in the lab. What are the pros and cons of both approaches?

l.38ff. The Module descriptions should be part of the main part of the article, since they do not represent method but rather provide key content required to understand the assessment. It would be important that:

(a) .. it is explained in the methods how the experiments are conducted and provide protocols and lesson plans as supplementary materials. What are typical results that students obtain from their experiments? What are the learning outcomes deduced?

(b) .. pictures of the process and example graphs are shown that illustrate the activities.

(c) .. the teaching materials are made available online so that others can join in. I recommend figshare.com as one free repository providing a DOI, but others might work as well.

l.99: The forms used for surveys should be made available as supplementary materials.

Results: Statements here are too vague since the reader is given too little information of how data were obtained. For example, how did authors determine that students had a better understanding of the items listed in the graphs (see comment on survey forms above). Many items show no real difference between ‘before’ and ‘after’, but this is not properly explained.

l.143: The info on the history of the initiative should be at start of the main section rather than the end. It describes strategy development and provides the background for the resource modules (as key outcome of the scientist-teacher collaboration) and the obtained data of their application.

The Discussion does not offer much. It should properly dissect the gains from this initiative and compare them to other projects or think about how the initiative could be further improved and expanded, potentially turned into a lasting effort.

Reviewer 2:

I would like to congratulate the authors on creating such a significant outreach programme which includes assessment of the success of the programme for their target audience. I find their main conclusions- which is that engagement with teachers who are expert in understanding the student curriculum in order to align learning needs, to be a universal and important (and often overlooked) message within science outreach and engagement. And likewise highlighting the value of science outreach/engagement professionals and the value of pre and post event assessment.

I have some suggestions/improvements, which I think would make the paper more internationally relevant.

1. There is no mention of funding in the paper. How was the Outreach funded and was there contribution from schools/students? Primarily I am interested in if schools/students self-select by who could cover costs associated with the Outreach (i.e. time for teachers to undertake professional development, consumables associated with Outreach etc)

2. Did the authors find an association with schools that undertook Outreach and measures of deprivation? Did measures of improved science proficiency correlate with these measures?

3. Was there a reason to target ‘city schools’ and not, for example areas of low science capital such as rural areas? I am unsure if by ‘City Schools’ this is a term associated with areas of deprivation or low science capital. In my country (the UK), pupils in schools in cities often have higher ‘science capital’ because of their access to museums, science centres etc. Clarification would be useful to ensure the paper is internationally understandable

4. The authors mention tailoring the Outreach by season (i.e. Fall, Spring etc) because this is when certain parts of the student curriculum are in action. I think the season is irrelevant and not very useful for understanding the paper in context of other countries- it would be better to explain how the Outreach is tailored by the curriculum.

5. what age are the students targeted? why this age? is there a difference in outcomes for students of different ages?

6. there is some discussion of the value to the outreach practitioners (i.e. post-grads, post-docs etc). Was there any training for the practitioners? Would it be appropriate in the future to also assess their outcomes, as this programme, is in fact a partnership in which the scientists should also benefit.

## Author Response

We thank Dr. Erskine, Dr. Prokop, and Reviewer #2 for helpful, constructive feedback on our manuscript. While the reviewers agreed that our manuscript “nicely encompasses central tenets of high-quality science outreach and public engagement with research”, all expressed concerns about the depth of our article. Further, the reviewers indicated several areas in the text where additional information would make our paper more useful as a template for outreach development. Below, we detail the modifications we have made in response to these comments. We believe that we have addressed the primary concerns of all reviewers and significantly strengthened our manuscript.

Dr. Prokop (Reviewer 1)

Dr. Prokop described our outreach initiative as “impressive” and noted that he would “love to see this work published”. He did, however, raise major concerns about the structure and depth of the article, which we address below.

“The introduction is far too short and lacks important information, as clearly illustrated by the fact that there are no references at all.”

We thank Dr. Prokop for suggesting points that could be discussed in our manuscript introduction. We have now significantly expanded our introduction, added citations and addressed each of his points.

“Why is there a need for science education?”

We have now discussed the need for diversification of the scientific workforce in the United States, a primary motivator for this outreach effort (Lines 22-28). We go on to describe the specific challenges faced by city school districts in the United States, which are for historic reasons primarily minority, and where spending on a per student basis is significantly lower than spending in suburban schools (Lines 29-42). We have also edited the introduction to provide important additional context: our university is in a large city (Philadelphia), so serving city schools specifically reflects service to our community. We draw the reader’s attention specifically to the way that nation-wide trends in science identity and science workforce participation are reflected in the School District of Philadelphia (Lines 33-56).

“Given the curriculum, what are the short-comings and how can scientists help?”

We are motivated by the fact that there are multiple barriers to entry for minority groups in the sciences, and our revised manuscript addresses this issue in several places. First, students educated in under-resourced schools may have significant knowledge gaps compared to their peers at better-funded institutions. Scientists can help by providing hands-on experiences that reinforce the science curriculum and by providing a mechanism for public school districts to take advantage of the resources of better-funded colleges and universities in their locales (Lines 58-65; 82-85). Furthermore, connection to scientists in the community may promote science identity (Lines 85-88).

“What aspects of the curriculum are being targeted and why?”

We have now made the point that the biology curriculum can be particularly challenging and that other studies have shown that students have benefitted significantly from hands-on biology instruction (Lines 47-56). We also mention the intersection of our neurogenetics research program with the high school curriculum; namely, as fly biologists, we are well-positioned to reinforce the experimental design and genetics curricula (Lines 68-70; 91-95).

“Why does Drosophila provide a good means to fulfil this task? What other initiatives are already out there, and how does this initiative complement existing resources and strategies? [This]would offer opportunities to compare/contrast the different approaches: sending scientists as teaching assistants into schools versus having teachers in the lab. What are the pros and cons of both approaches?”

We now discuss the use of Drosophila in the classroom, including other outreach efforts that have successfully used flies, in the Introduction (Lines 70-76). While we think an extensive comparison to existing strategies is beyond the scope of this manuscript, we agree that it would be an interesting area of research and discussion.

“It is never made clear how the activities link to the authors’ own research.”

We thank Dr. Prokop for raising this point. In the initial draft of the manuscript, we eliminated direct references to our research in light of eNeuro’s double-blind peer review process. We have now restored these references because we think that they are important for understanding the context of the program. Please refer to lines 67-70 and 221-227 for lines addressing the relationship between the work in our lab and the outreach program.

“The Module descriptions should be part of the main part of the article, since they do not represent method but rather provide key content required to understand the assessment.”

We agree with Dr. Prokop’s assessment and have moved the module descriptions out of the Methods section into an independent section in the main part of the article (Lines 183-261). We also describe the phases of module development in a distinct “Approach” section (Lines 120-182).

“It would be important that...it is explained in the methods how the experiments are conducted and provide protocols and lesson plans as supplementary materials. What are typical results that students obtain from their experiments? What are the learning outcomes deduced?”

We thank Dr. Prokop for this helpful suggestion. We have added additional details, including two new figures (now Fig.1 and Fig.2), to support our explanation of the modules (Lines 183-236). These figures highlight typical results obtained from both experiments. We have also provided the questions asked in our pre- and post-module surveys as a Supplemental Table (Supplemental Table 1).

”.. pictures of the process and example graphs [should be] shown that illustrate the activities.”

We believe that new Figures 1 and 2 will also address this concern and thank Dr. Prokop for this suggestion.

”...the teaching materials [should be] made available online so that others can join in.”

We have added new Supplemental Information containing lesson plans and workbooks for both modules (Supplemental Information 1 and 2). We have also added text in the Methods section asking readers interested in replicating our study to contact one of our co-authors who has many years of experience building sustainable programming (Lines 101-104).

“The forms used for surveys should be made available as supplementary materials.”

At Dr. Prokop’s suggestion, we have provided the questions asked in our pre- and post-module surveys as a Supplemental Table (Supplemental Table 1).

“Results: many items show no real difference between ‘before’ and ‘after’, but this is not properly explained.”

We thank Dr. Prokop for raising this important point. We now include a discussion of the possible explanations for the minimal changes we observed in some content areas (Lines 275-283).

“The info on the history of the initiative should be at start of the main section rather than the end. It describes strategy development and provides the background for the resource modules (as key outcome of the scientist-teacher collaboration) and the obtained data of their application.”

We find Dr. Prokop’s critique helpful and have moved the information into an “Approach” section (described above).

“The Discussion does not offer much. It should properly dissect the gains from this initiative and compare them to other projects or think about how the initiative could be further improved and expanded, potentially turned into a lasting effort.”

In our revised manuscript, we address the future of our program in two places. First, in describing our approach, we describe a plan for a third phase that would allow us to expand our reach within our existing budget (Lines 170-181). Further, in the Discussion, we raise the funding and personnel hurdles to sustainable outreach programming alongside our plans to address them (Lines 315-325).

Reviewer 2

We appreciate Reviewer 2’s acknowledgement that we have created a “significant outreach programme which includes assessment of the success of the programme for [our] target audience” and made several “often overlooked” points regarding the importance of teacher and outreach professional partnerships in outreach efforts. Reviewer 2 did, however, express concern that our article was not formulated to be internationally relevant. We believe that the changes to the manuscript that we describe below address these concerns.

“There is no mention of funding in the paper. How was the Outreach funded and was there contribution from schools/students? Primarily I am interested in if schools/students self-select by who could cover costs associated with the Outreach (i.e. time for teachers to undertake professional development, consumables associated with Outreach etc)”

We agree with Reviewer 2 that this is a critical consideration for our outreach program structure. Many of the mentions of funding, which were initially stripped from our manuscript to preserve the integrity of eNeuro’s double-blind peer review process, have been restored. Specifically, we direct Reviewer 2 to the following lines in the manuscript:

- Teachers who conduct research and develop outreach modules in our lab are compensated (Lines 124-125; 136-137)

- Teachers who participate in professional development associated with our outreach efforts (for example, attending training sessions) receive credit toward their state-mandated professional development requirements (i.e. “Act 48”; lines 158-159).

- This project was initially conceived of as a “broader impacts” component of a National Science Foundation grant (Lines 132-133). Funding from the National Science Foundation and matching funding from our home department allowed us to hire a designated outreach educator (Lines 160-163).

“Did the authors find an association with schools that undertook Outreach and measures of deprivation? Did measures of improved science proficiency correlate with these measures?”

We thank Reviewer 2 for these interesting questions. In the United States, because property taxes directly fund schools in the community, under-resourced schools can be identified in part by the proportion of students who receive free and reduced cost lunch. We have amended our manuscript to mention that all students in the School District of Philadelphia are eligible for free and reduced cost lunch (Lines 33-35).

“Was there a reason to target ‘city schools’ and not, for example areas of low science capital such as rural areas? I am unsure if by ‘City Schools’ this is a term associated with areas of deprivation or low science capital. In my country (the UK), pupils in schools in cities often have higher ‘science capital’ because of their access to museums, science centres etc. Clarification would be useful to ensure the paper is internationally understandable.”

We appreciate the importance of making our manuscript internationally legible and thank Reviewer 2 for this feedback. In our introduction, we now discuss the historic problem of underfunding city schools, which because of historic housing policy in the United States, serve a higher proportion of minority students than suburban and rural schools (Lines 22-42). Because our university is in a major city, we are well-positioned to serve city students.

“The authors mention tailoring the Outreach by season (i.e. Fall, Spring etc) because this is when certain parts of the student curriculum are in action. I think the season is irrelevant and not very useful for understanding the paper in context of other countries- it would be better to explain how the Outreach is tailored by the curriculum.”

We thank Reviewer 2 for this insight. We have changed our discussion of outreach modules so that they reference the topics covered by the curriculum at the time of the outreach experience (Lines 247-249) except where we felt that emphasizing that the data was gathered over a single school year was important (namely, in Figure 4).

“What age are the students targeted? Why this age? Is there a difference in outcomes for students of different ages?”

Our program targets students in high school biology, which is where we feel our research area (Drosophila neurogenetics) best aligns with the science curriculum (Lines 68-70). While agree with Reviewer 2 that it would be interesting to consider outreach programs for students of different ages, this was not a component of the current study.

“There is some discussion of the value to the outreach practitioners (i.e. post-grads, post-docs etc). Was there any training for the practitioners? Would it be appropriate in the future to also assess their outcomes, as this programme, is in fact a partnership in which the scientists should also benefit.”

We enthusiastically concur with Reviewer 2 on this point, and we hope to assess the outcomes for practitioners in a future project (Lines 329-344). While classroom visits are led by an experienced outreach educator (Lines 160-163), we have not formalized a training process for other outreach practitioners at this time.
