# Supplementary material for: DrosoPHILA: A Partnership between Scientists and Teachers That Begins in the Lab and Continues into City Schools
Source: eNeuro. 2023 Feb 13;10(2):ENEURO.0263-22.2022. doi: 10.1523/ENEURO.0263-22.2022 (PMC9927510; doi:10.1523/ENEURO.0263-22.2022)
Supplement: Extended Data 1 — Lesson plans for Flies on Ice. Download Extended Data 1, ZIP file. [file enu-eN-NWR-0263-22-s04.zip › Extended data 2.pdf]

# Flies on Ice

DrosoPHILA

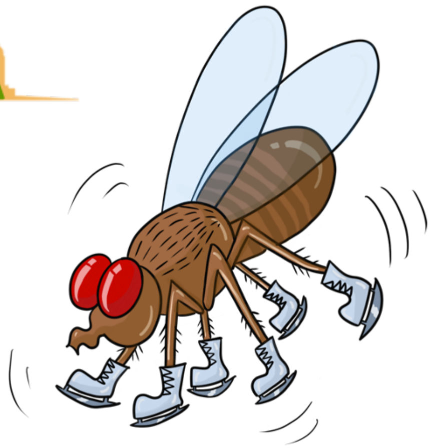

Using *Drosophila* as a model organism to teach methods of experimental design and investigate the nervous system.

Objective:
SWBAT design an experiment, follow a protocol, collect and analyze data IOT investigate a scientific question

Table of Contents:

I. General Overview and suggested timeline …………………..…. p. 2
II. Connections to the Biology Curriculum …………………….….. p. 3
III. Background Information ……………………………………… p. 4
IV. Specific Suggestions for Implementation ……………….......... p. 5
V. Extension Activities …………………………………………..... p. 8
VI. Standards Alignment …………………………………………... p. 9
VII. Day 2 Procedure ………………………………………………. p. 10

**I. General overview and suggested timeline:**

We recommend taking three days to perform this lesson; but there are many options available to shorten or extend it as needed. We also suggest teaching this unit after students have learned about cell membrane components and their function, as an introduction to exocytosis, while also reviewing scientific inquiry and mechanisms of enzyme action. However, a simplified version of the unit can be used at the beginning of the year or if you are teaching this lesson to a lower grade level. A sample schedule is provided here:

Day 1 - Introduce flies and the phenomenon of knocking out flies on ice; Students define variables and plan their procedure to answer the question: How does the amount of time flies spend on ice affect their activity?

Day 2 - Students conduct their experiment and collect data.

Day 3 - Students analyze the results from the experiment, discuss the molecular explanation for paralysis/ cell communication, and draw conclusions.

***One or two days prior to beginning this unit, administer the provided pre-assessment. Students should not write their name on the survey. Rather, the teacher should assign them a number that they will use to identify themselves again on the post-assessment, provided either at the end of class on Day 3 (if there is time) or the following day.*

## II. Connections to the Biology Curriculum

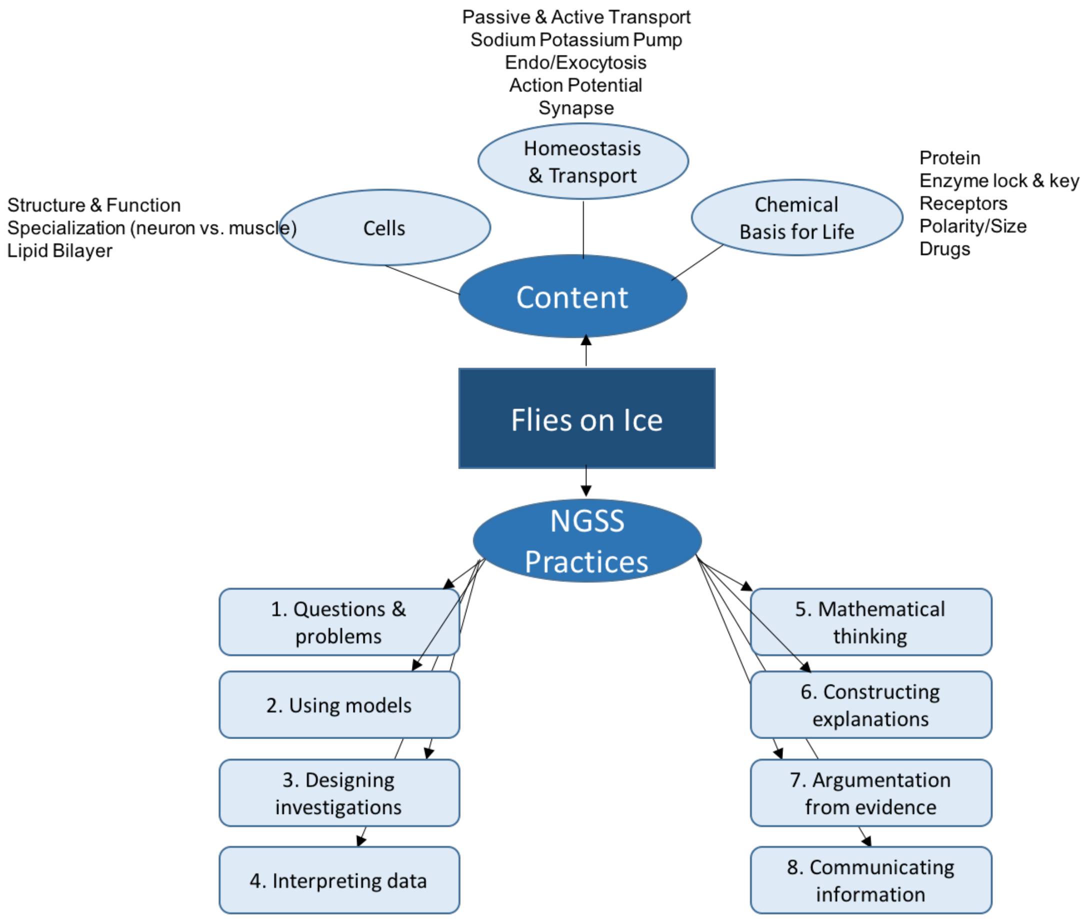

## III. Background Information:

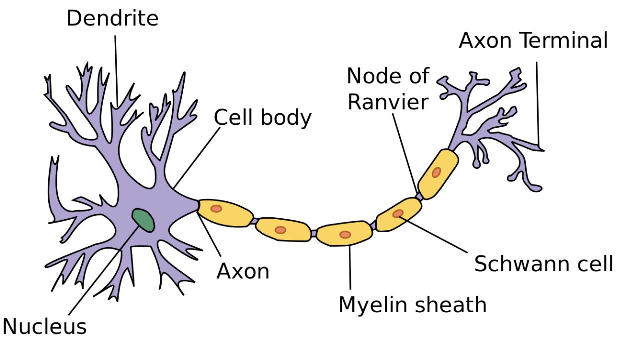


Axons are the wires of the nervous system (see the figure to the right). Electrical signals travel down the axon and cause synaptic vesicles within the axon terminal to release neurotransmitters that bind to receptors on the postsynaptic cell. This binding of neurotransmitter elicits a reaction, such as muscle contraction (if the postsynaptic cell is a muscle cell) or the firing of the downstream neuron. The neurotransmitters are then released (from the postsynaptic cell) back into the synaptic cleft and taken up by the presynaptic cell to be used again (see the figure below).

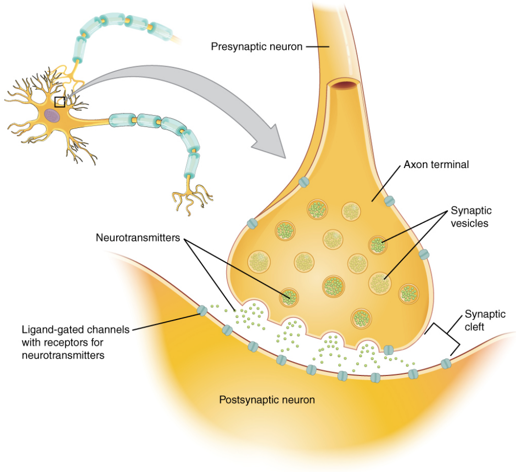


This process is very sensitive to temperature, so if the neurons and muscles get too cold, they stop working. The cold temperature prevents the enzymatic activities required for membrane invagination and proper reuptake of the neurotransmitter. Once the flies are taken off ice and the body temperature warms up, enzymatic activity resumes, allowing the presynaptic cell to reabsorb the neurotransmitters so that it can “fire” again.

Generally, it takes approximately 1-3 minutes for the flies to warm up and resume circuit activity. This is regardless of the total length of time the flies were knocked out, since the fly can only become as cold as the ice. This holds true as long as the flies aren’t kept on the ice for 24+ hours; this length of time would ultimately destroy the cells and kill the fly. A good indication for recovery is looking for the first sign of wing movement. We found that most flies take, on average, 2 minutes for their wings to begin twitching after being on ice. However, you should encourage the students to discuss amongst themselves what they feel the criteria should be for “recovery”.

To help students understand this process of how flies become immobile when on ice, consider making an analogy with nerf guns. The foam darts are messages. You (the neuron) can send the message to the person at the other end of the room by shooting the dart with your nerf gun. In order to continue sending messages, however, you have to reload your nerf gun

with darts. But if you can’t reload (because you are out of darts, for example), your messages don’t get sent.

## IV. Suggestions for Implementation:

| **Day 1 Agenda** | |
|---|---|
| Student workbook pages 2-10.<br><br>Slide deck slides 1-13.<br><br>Materials needed per group of 4 students:<br>● 1 solo cup filled with crushed ice**<br>● 2 empty vials<br>● 2 food vials with flies<br>● Magnifying glass (optional)<br><br><br>Key Vocabulary:<br>● Genes<br>● DNA<br>● Proteins<br>● Enzymes<br>● Nervous System<br>● Neuron<br>● Endotherm/ Warm-blooded<br>● Ectotherm/ Cold-blooded<br><br>***How to break ice cubes, if you don’t have easy access to crushed ice:*<br>*(1) Make the ice cubes in your freezer. Put* | ● Introduce fruit flies (*Drosophila melanogaster*) and why the are important for biomedical research (have you seen this organism before? where/in what context? etc…).<br>● Briefly discuss the concept of model organisms, their value for science research, the nervous system, and the benefits of *Drosophila* as a model system. Students answer the prompts on page 4 and 5.<br>○ A fact sheet for more information on model organisms: https://www.nigms.nih.gov/Education/Pages/modelorg_factsheet.aspx<br>○ A fact sheet for more information on fruit flies: ww2.biol.sc.edu/~elygen/.../Drosophila%20melanogaster%20Fact%20Sheet.docx<br>● Review the fruit fly life cycle and have student observe a vial with various stages present. Students fill out page 7.<br>● Briefly compare endotherm responses to cold temperatures and compare with ectotherms. Students answer the prompt on page 9.<br>● Demonstrate how to transfer flies from one vial to another.<br>○ Here is a link to show you how to do this: https://www.youtube.com/watch?v=geNpxD_QNu4<br>● Explain to students that they have about 6 minutes to make some preliminary observations of the flies at room temperature and when placed on ice. Tell students that to see the fly response on ice, they should push the flies from the food vial into the empty vial. |

| | |
|---|---|
| *them in ziploc bags and gently hit or crush them with a rolling pin.*<br>*(2) Purchase a large bag of ice from the grocery store. Hold the bag on its side and drop it straight down on the floor. Pick up the bag and do the same thing on the other side.*<br>(3) Crushing ice in a blender or snow cone maker also works. | ● Pass out the materials (1 cup of ice, 2 food vials with flies and 2 empty vials) to each group of 4 students.<br>● After about 6 minutes, every group shares one observation they made about their flies.<br>● Students push the flies back into the food vials and bring them up to you. You will use these same flies for Day 2.<br>● Propose the following question to the students for Day 2: **How does the amount of time flies spend on ice affect their activity? In other words, if the fly stays on the ice for a longer period of time, does it take a longer time for it to regain activity?**<br>● If there is time at the end of class, have students create a protocol to test 1, 5, and 10 minutes. Collect the student workbooks. Read over the student protocols and pick out the best one for the rest of the class to follow. In the event that none of the groups create a clear-cut protocol, you can photocopy and distribute the one we developed (found at the end of the teacher notes). If multiple groups have created acceptable protocols, they may follow their own as long as they have standardized their definition of 'recovery'. |

| **Day 2 Agenda** | |
|---|---|
| Student workbook pages 11-15.<br><br>Slide deck slides 14-23.<br><br>Same materials as Day 1<br><br>Key Vocabulary:<br>● Hypothesis<br>● Independent | ● In groups (or individually), students work on the student workbook. These pages of the workbook will guide students through how to design the experiment in order to answer the question. Students fill out page 11.<br>○ Lead a discussion on how to define and then standardize 'recovery time'. Also discuss why this is an essential factor to standardize.<br>○ Specify that the experimental groups for time on ice will be 1, 5, and 10 minutes.<br>● If collected previously, distribute the student-designed protocol chosen from Day 1 (or the protocol found with the student handout). Review the steps with the students. |

<table>
<tr>
<td>variable<br>● Dependent variable<br>● Protocol<br>● Controlled variable</td>
<td>During this time, review what it means to “regain activity”. Emphasize that it is really important to maintain class-wide consistency so that a valid conclusion can be drawn from the experimental data!<br>● Students conduct the experiment and collect their data on page 14<br>● Once they have finished collecting data, students should:<br>  ○ Share the data with you so that you can create a class data set<br>● At the end of class:<br>  ○ The ice can be thrown down the sink, or saved in the science freezer for future experiments.<br>  ○ The flies can be pushed into new food vials, to be used in future experiments. If you need to dispose of them for whatever reason, freeze them for at least 24 hours in the science freezer before throwing them away.<br>  ○ Facilitate a class discussion about sources of error and intervening variables (variables that could not be controlled but may have affected results). Ask students to propose improvements to the experiment.</td>
</tr>
</table>

<table>
<tr>
<td colspan="2">Day 3 Agenda<br>**There are different ways to approach this third day, depending on the skill level and technology available to your students.</td>
</tr>
<tr>
<td>Student workbook pages 15-18.<br><br>Slide deck slides 24-34.<br><br>Key Vocabulary:<br>● Enzyme<br>● Axon<br>● Synapse<br>● Neurotransmitter<br>● Presynaptic</td>
<td>● Students use their data or class averages from page 14 to create a graph on page 15.<br>● Discuss what happens at the cellular level when a fly is put on ice (action potential triggers release of neurotransmitters by exocytosis into synapse cleft, ligands bind with receptors to initiate a response)</td>
</tr>
</table>

| | |
|---|---|
| • Postsynaptic | • Students write a claim, supporting evidence and reasoning for their experiment on page 17. Students can read page 18 for additional information about neurons and the effect of temperature. |

**V. Extension activities:**

- Discussing the effects of venom/toxins/drugs on the inhibition of synaptic function
  - Animals like the puffer fish, blue-ringed octopus and poison dart frog have tetrodotoxin that block the generation and propagation of action potentials by preventing the movement of sodium ions across cell membranes.
    - National Geographic video on the puffer fish (https://www.youtube.com/watch?v=OkXhC7yzISI)
- Discussing how drugs like SSRIs (Selective Serotonin Reuptake Inhibitors) function to inhibit reuptake of a specific neurotransmitter
  - Khan Academy explanation https://www.khanacademy.org/science/health-and-medicine/mental-health/depression-and-related-disorders/v/treating-depression-with-antidepressants
- Discussion on human nervous system disorders, such as:
  - Parkinson's Disease (https://www.youtube.com/watch?v=VIEUEV9wlyI)
  - Multiple Sclerosis (https://www.youtube.com/watch?v=Naecv3h868c)
- Discussion on the use of flies as model organisms
  - https://droso4schools.wordpress.com/organs/
- Learning the mechanism of action potentials as they relate to signaling the release of neurotransmitters.
  - Khan Academy on what is an action potential (https://www.khanacademy.org/science/health-and-medicine/nervous-system-and-sensory-infor/neuron-membrane-potentials-2014-03-27T17:58:17.207Z/v/neuron-action-potential-description)
  - BBC Bitesize Science http://www.bbc.co.uk/schools/gcsebitesize/science/add_ocr_pre_2011/brain_mind/informationrev2.shtml
  - Dros4Schools resources on understanding synapses and action potentials https://droso4schools.wordpress.com/l3-neurons/#4 https://droso4schools.wordpress.com/l3-neurons/#7

## VI. Standards Alignment

Next Generation Science Standards

HS. Structure and Function

Students who demonstrate an understanding can:

*HS-LS1-3*. Plan and conduct an investigation to provide evidence that feedback mechanisms maintain homeostasis.

Pennsylvania State Science Curriculum

Standard 3.1.B.A9

- Identify questions and concepts that guide scientific investigations.
- Know that both direct and indirect observations are used by scientists to study the natural world and universe.
- Evaluate experimental information for relevance and adherence to science processes.
- Interpret results of experimental research to predict new information, propose additional investigable questions, or advance a solution.

Pennsylvania Biology Keystone Assessment Anchors

BIO.A.1: Basic Biological Principles

*BIO.A.1.2:* Describe relationships between structure and function at biological levels of organization.

*BIO.A.2.3*: Explain how enzymes regulate biochemical reactions within a cell

BIO.A.4: Homeostasis and Transport

*BIO.A.4.1*: Identify and describe the cell structures involved in transport of materials into, out of, and throughout a cell

**Day 2: Standard Flies on Ice Procedure**

1. Gather materials: 2 cups of ice, one food vial of 5-7 flies, one empty vial, stopwatch

2. Push the flies from the 2 food vials into the 2 empty vials ("tap, tap, flip"). Label 1 vial '1, 2, 5 min' and the other one '10min'.

3. Put the empty vials (now with flies) on ice. Make sure that the ice covers the vials entirely! Set 1 timer for 10 minutes and set another for 1 minute.

4. After one minute, take one vial out of the ice.

5. Leave it on your table. Wipe down the condensation once with a tissue or paper towel. Keep a close eye on the flies!

6. Once a fly begins to ____(whatever your group decides is the sign of regaining activity)_____, record in the chart below the time it took the fly to do this motion.

7. Once all the flies have regained activity, wait another 30 seconds before repeating the experiment for 2 minutes, and 5 minutes.

8. When the 10 minute timer goes off, repeat the procedure for that vial.

9. Record your data on your sheet and contribute your data to the class data sheet.

10. Push the flies from the empty vials back into the food vials ("tap, tap, flip"). Return all supplies.
