# Supplementary material for: DrosoPHILA: A Partnership between Scientists and Teachers That Begins in the Lab and Continues into City Schools
Source: eNeuro. 2023 Feb 13;10(2):ENEURO.0263-22.2022. doi: 10.1523/ENEURO.0263-22.2022 (PMC9927510; doi:10.1523/ENEURO.0263-22.2022)

FALL 2022

FLIES ON ICE

A DROSOPHILA INVESTIGATION

Name ______________________ **Student ID** ______________________

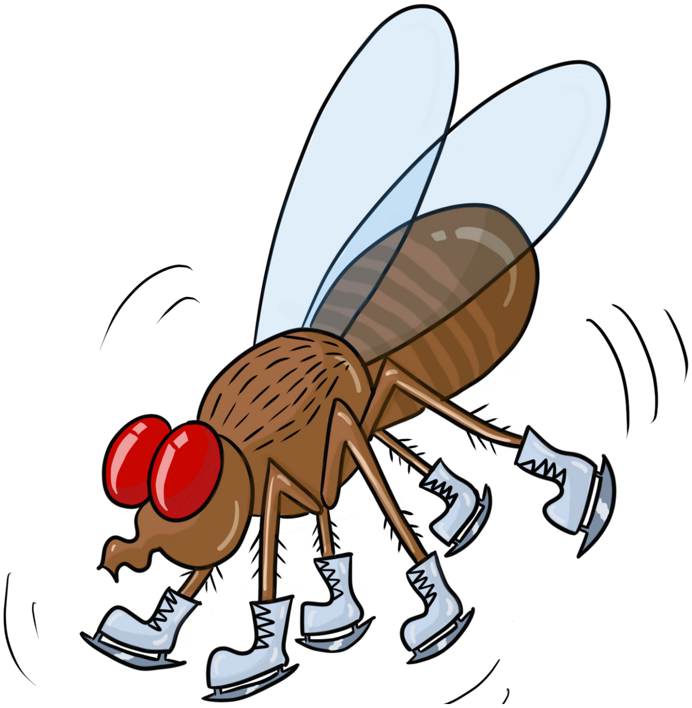

Perelman
SCHOOL OF MEDICINE
UNIVERSITY of PENNSYLVANIA

IOS 1853719

TABLE OF CONTENTS

3 WELCOME

Meet the scientists and educators behind DrosoPHILA

4 WHAT IS A MODEL ORGANISM?

Learn how scientists use fruit flies to understand human biology

5 WHAT IS THE NERVOUS SYSTEM?

Learn why scientists study how neurons work

6 INVESTIGATION, DAY 1

Learn about fly behavior at different stages of the fly life cycle and make preliminary observations about how flies respond to being placed on ice

11 INVESTIGATION, DAY 2

Develop a hypothesis for how flies' recovery time will be affected by flies' time on ice,test that hypothesis, and graph your data.

16 INVESTIGATION, DAY 3

Interpret your data and compare your results to pooled data in your class

Front and Rear Illustrations by
Kelly Sullivan.

DrosoPHILA
IOS 1853719

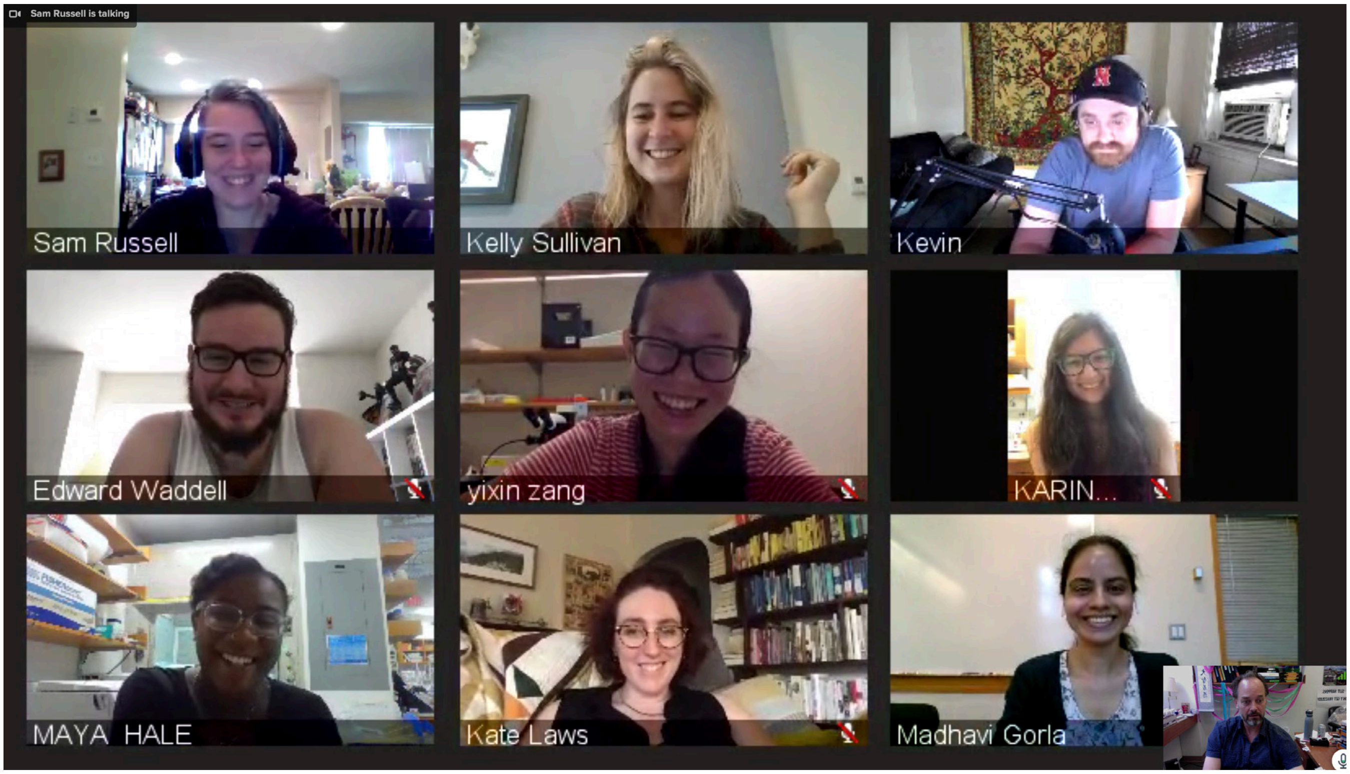

Members of the Bashaw lab gather virtually in August 2020

WHO WORKS IN A LAB?

PRINCIPAL INVESTIGATOR Manages all projects in the lab, applies for funding, and sometimes teaches classes. Has a Ph.D. and often postdoctoral research experience.

POSTDOCTORAL RESEARCHER Develops an independent research program as an apprentice to the principal investigator and sometimes teaches classes. Has a Ph.D.

GRADUATE STUDENT Works on an independent research project under the supervision of the principal investigator while taking classes. Can be pursing a Ph.D. or a Master's degree. Has at least a Bachelor's degree. Master's programs are usually 1-2 years long. Ph.D. programs can take 5 years. A Master's degree is not required to pursue a Ph.D.

RESEARCH ASSISTANT Supports projects in the laboratory and sometimes develops their own project. Research assistant positions can be pursuing a Bachelor's degree. People who hold an Associate's, Bachelor's, Master's degree, or Ph.D. can also work professionally as research assistants, specialists, or technicians.

LAB MANAGER A professional position that typically requires at least a Bachelor's degree. Lab managers are responsible for ordering materials, maintaining equipment and space, and scheduling meetings. They are usually familiar wtih the research in the lab and may assist with research.

WHAT IS A MODEL ORGANISM?

What flies can tell us about human biology

In the Bashaw laboratory, we use fruit flies as **model organisms** for our scientific research. A model organism is a non-human organism that is extensively studied to provide insight into the biological processes of another organism, particularly humans.

Model organisms are important for us to increase our understanding of basic biological processes like how different proteins function as well as our understanding of diseases like cancer. Using model organisms, scientists can conduct experiments that we would not do in humans. Various different organisms are considered model organisms, like yeast, zebrafish, mice, frogs, and even fruit flies.

Fruit flies are one of the oldest and most widely-used model organisms. Importantly, fruit flies and humans share about 44% of their **genes**. Genes are sequences of nucleotides in our **DNA** that encode for specific **proteins**. Proteins are essential molecules, and have important functions like providing structure to our cells, transporting things around our bodies, and allowing our organs to function properly. One group of proteins, **enzymes**, accelrate chemical reactions in cells, which is critical to sustaining life. The similarity between fruit fly genes and human genes make fruit flies a great organism to use to understand human gene function, including how abnormal gene function contributes to disease.

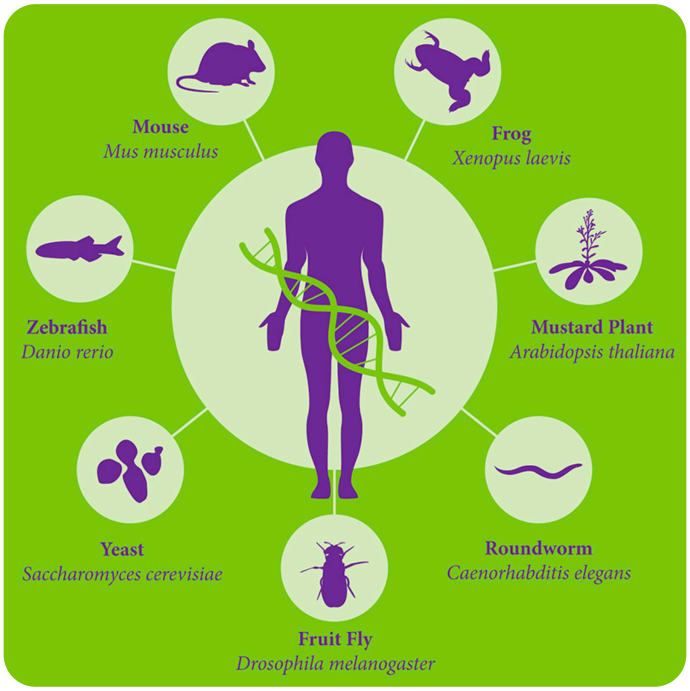

A number of different model organisms are used in biological research. Courtesy of the University of Iowa.

List three reasons why flies are great model organisms

1. ______________________________

2. ______________________________

3. ______________________________

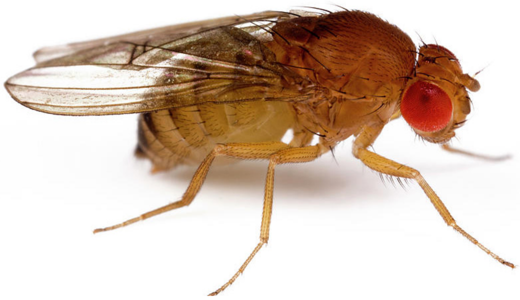

A female fruit fly. Image courtesy of Pascal Goetgheluck

WHAT IS THE NERVOUS SYSTEM?

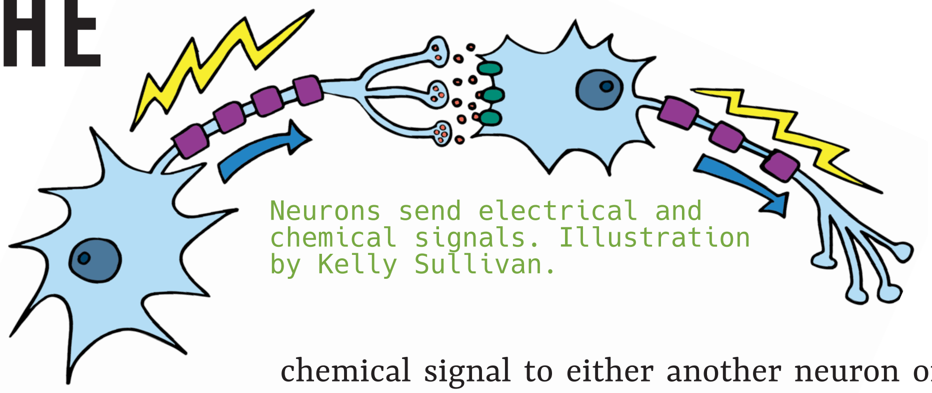

Neurons send electrical and chemical signals. Illustration by Kelly Sullivan.

Your **nervous system** influences almost all aspects of your life, from thinking, talking, and eating to moving, sleeping, and even dreaming.

Neurons are one of the major cell types in the nervous system. A **neuron** is a specialized cell which uses an electrical signal to send a chemical signal to either another neuron or to a specific target, like a muscle. That is how information is carried throughout your body.

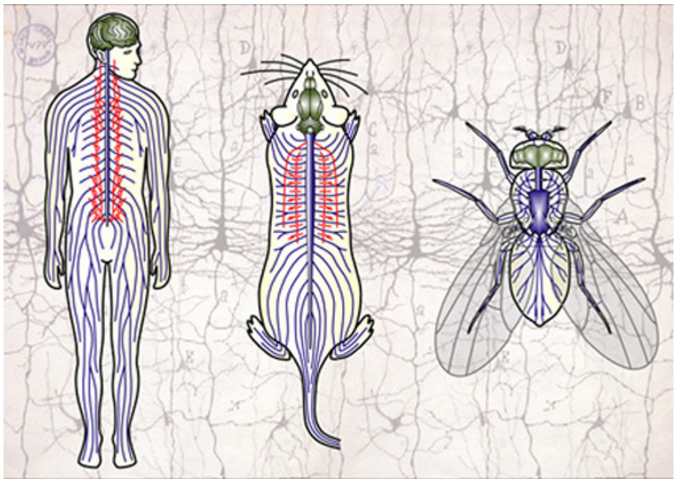

Like humans, model organisms (like mice and flies) have nervous systems. Illustration by Andreas Prokop.

For example, imagine you touch something hot with your hand. Your response will be to pull your hand away. Why is that your response? Cells in your hand become damaged by the heat and nearby neurons receive that signal. Those neurons send the information to the brain, where it is processed. The neurons in the brain send a signal back down to the muscles in your hand and arm to make your hand move away from the hot object. All of this happens in less than a second.

Like humans, flies have complex nervous systems made up of neurons that are connected to each other. It may surprise you, but flies actually do a lot of the things that humans do. Compared to humans, though, the fly nervous system is small, having many fewer neurons. This makes the fly nervous system easier to study and is yet another benefit to using flies as a model organism.

List three similarities between the nervous systems of humans and flies

1. ____________________

2. ____________________

3. ____________________

INVESTIGATION DAY 1

The life cycle of the fruit fly

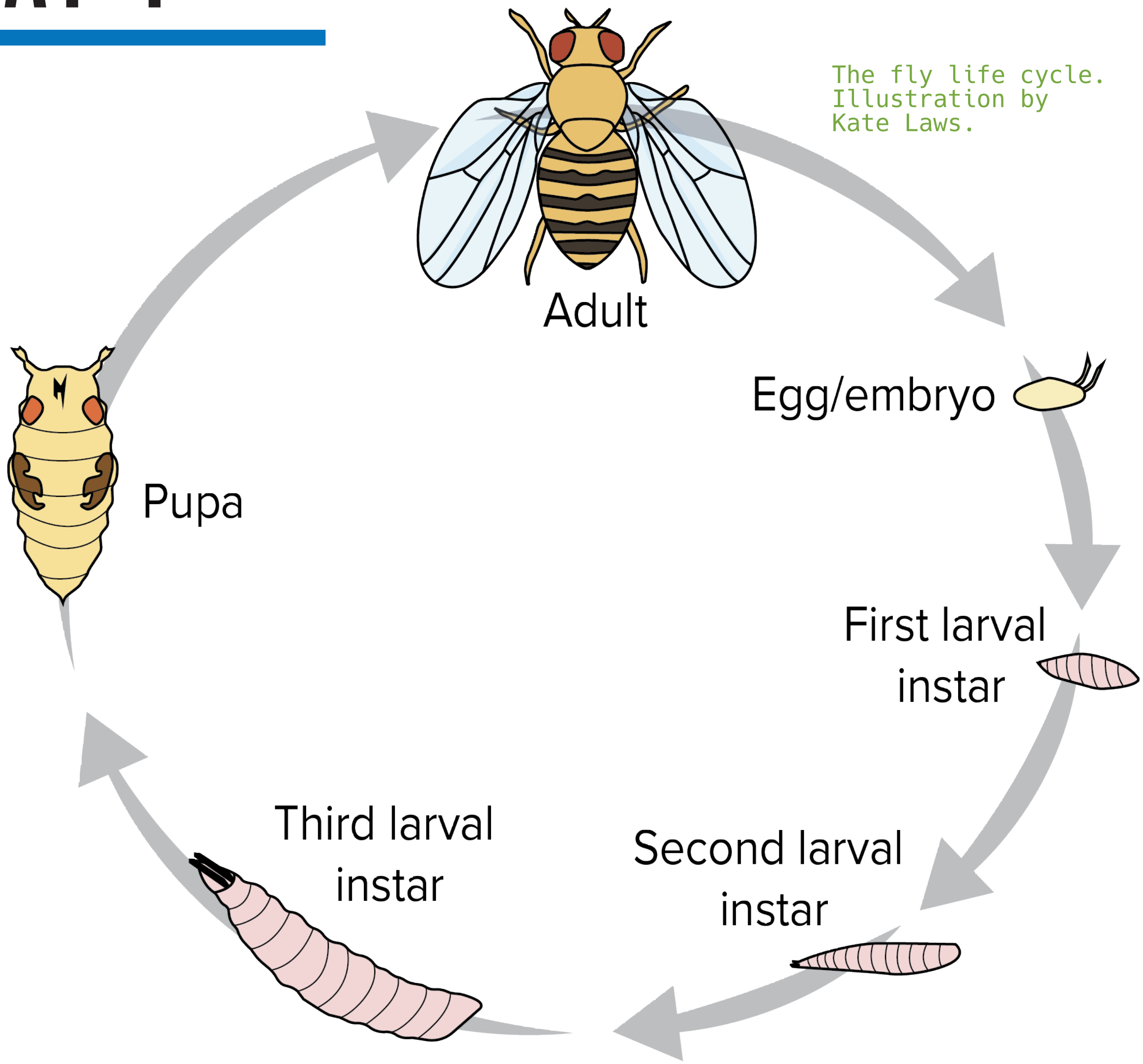

The fly life cycle. Illustration by Kate Laws.

The fruit fly has a complex life cycle that has four The fruit fly has a complex cycle with four distinct stages. When a female fly lays an egg, if that egg was fertilized by a male, an **embryo** develops. After about 24 hours, a **larva** hatches. A larva's primary function is to eat. As it eats, it undergoes three molts, or **instars**. At a certain point, the third instar larva receives a hormonal signal to stop eating and seek a place to form a **pupa**. During the pupal stage, the fly liquifies most of its body and builds the body parts of the **adult** fly, including legs, wings, and eyes. During each stage of this life cycle, flies have different roles, each of which is important for their ability to reproduce. Embryonic development sets up the body plan for the fly. The food consumed by the larva provides the energy needed to build new structures in the pupal stage, from which an adult emerges. The adult fly can walk, fly, see, eat, and reproduce.

Observe a food vial of flies. Use your observations of the different stages of the life cycle to answer the questions below

Which stages in the life cycle move? Which do not?

___

___

The eggs/ embryo are very small and not easily seen.
Make an inference: Where in the vial is the best location for fruit fly eggs/ embryos?

___

___

Where are the larvae in the vial?

___

___

How can you tell the difference between the larvae and pupae?

___

___

Where in the vial are the adult flies?
Identify two behaviors of the adults.

___

___

A CLOSER LOOK AT ADULT FLIES

Observe the male and female flies in the images on this page. Based on your observations, how can you tell male and female fruit flies apart?

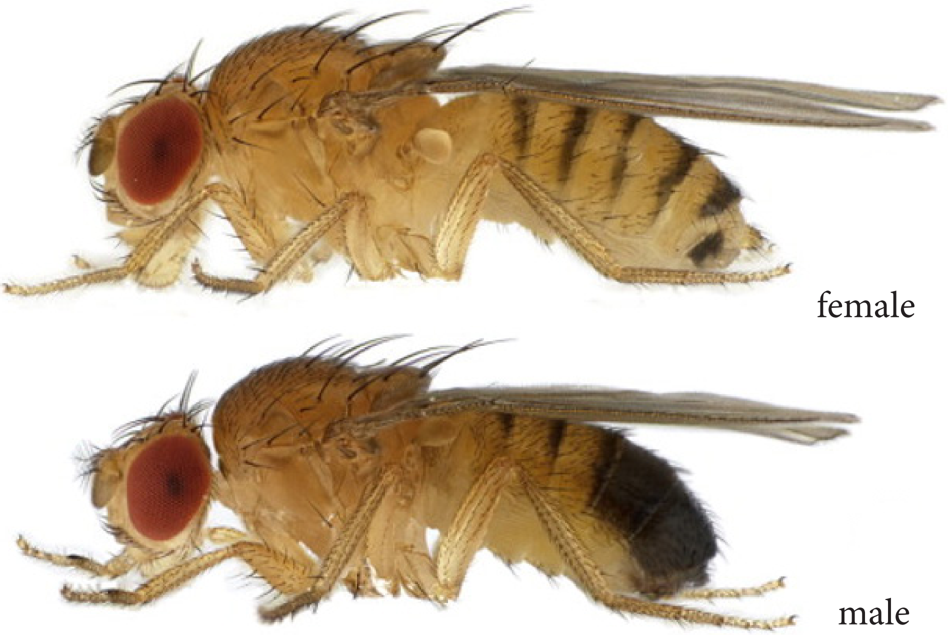

Adult female (top) and male (bottom) fruit flies.

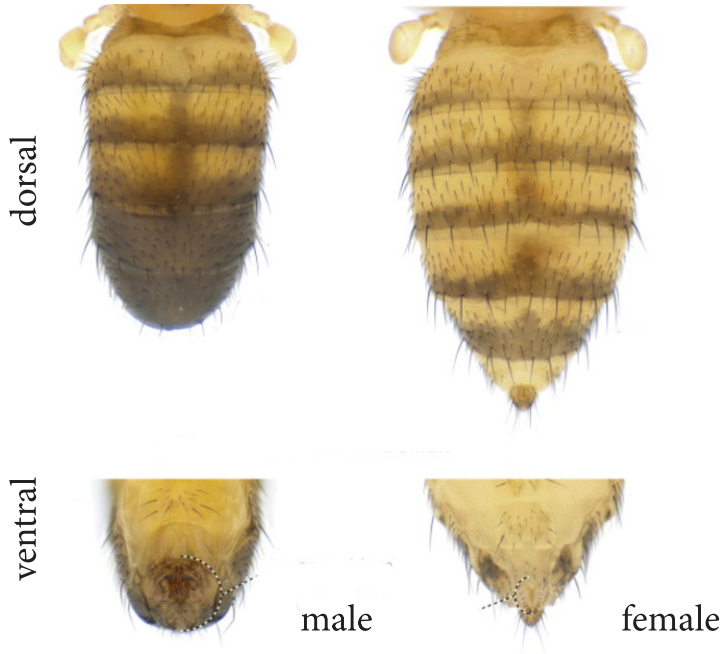

Abdomens of male and female flies viewed from the top (a dorsal view) or from the bottom (a ventral view).

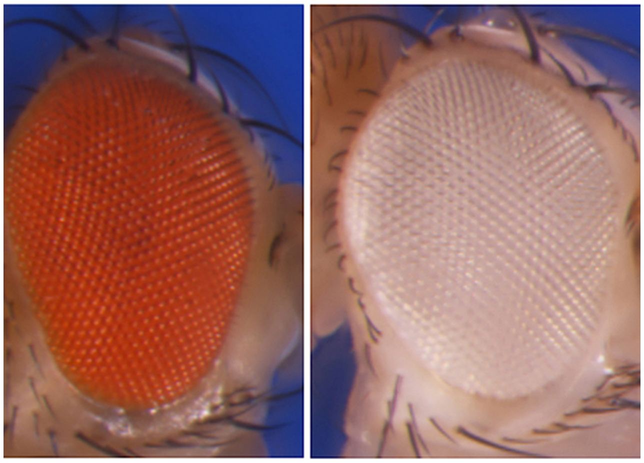

Red-eyed wild type flies (left) and white-eyed mutant flies (right). Photo from Indiana University.

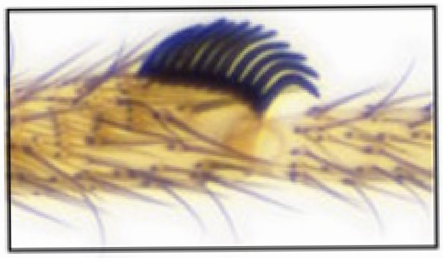

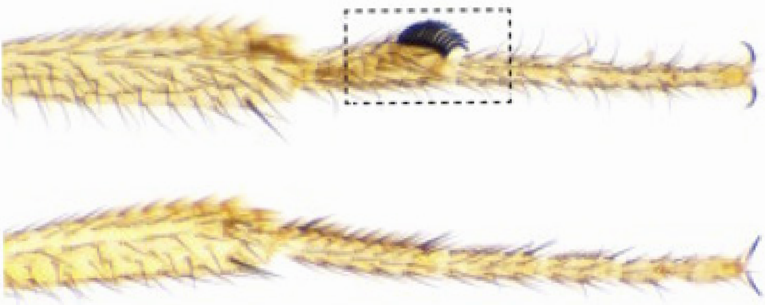

Legs of male (top) and female (bottom) fruit flies. The boxed in area on the male fly's leg is called a sex comb.

WHAT WILL HAPPEN TO FLIES' BEHAVIOR WHEN WE PUT THEM ON ICE?

While we have focused on the important similarities between humans and flies, there are also striking differences between them. For example, humans maintain a constant internal temperature of approximately 98.6 degrees Farenheight no matter what the temperature is around us. This is because humans, like other mammals, are **warm-blooded**, or **endotherms**. This means that we are capable of maintaining our own internal body temperature. This is why you sweat when you get hot and shiver when you get cold: both of these are homestatic mechanisms meant to stabilize your internal temperature. In contrast, flies, like most insects and reptiles, are **cold-blooded**, or **ectotherms**. This means that the body temperature of a fly matches the ambient temperature of its environment. As a result, we can dramatically alter the body temperature of a fly in ways that we cannot do with humans or other warm-blooded animals.

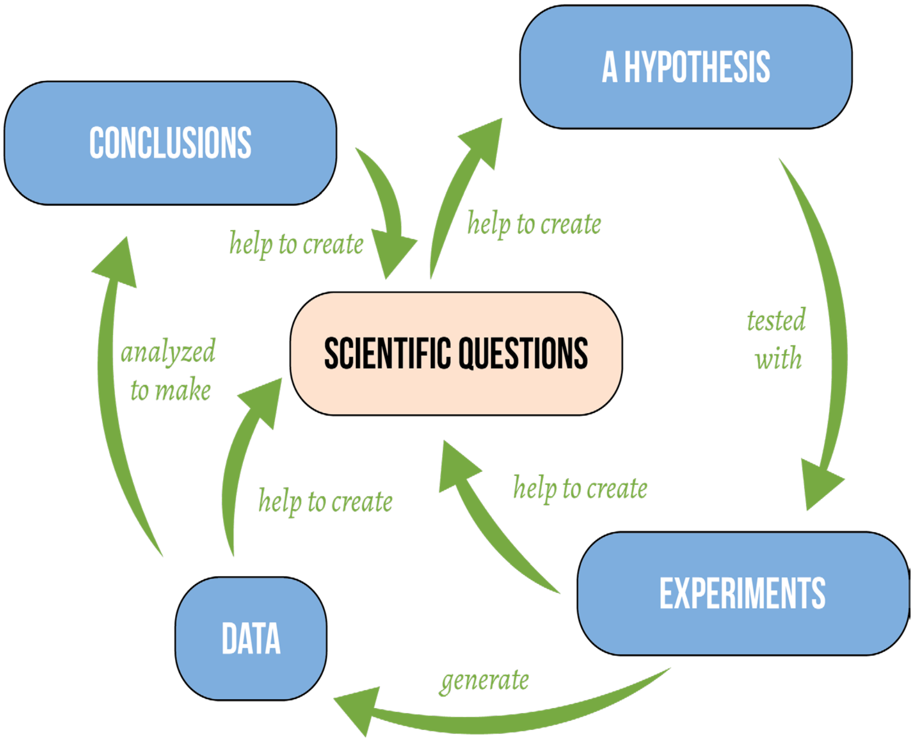

The scientific process

What happens to your body when you go outside on a cold day without a jacket on? How do you think this helps you maintain homeostasis, the equilibrium of your bodily functions?

PLANNING OUR EXPERIMENT

Scientific question: what will happen if we put flies on ice?

Hypothesis: IF we put flies on ice, THEN they will ______________________________

____

____

____

PRELIMINARY OBSERVATIONS

Below record observations about fly behavior under different conditions.

Room temperature ~75 degrees F	*Immediately after ice* below 32 degrees F	*30 seconds-2 minutes after ice* returning to ~75 degrees F

INVESTIGATION DAY 2

How does time on ice affect fly recovery time?

Based on your observations from yesterday, how would you define "recovery" for a fly that has just been taken off of ice?

____

____

____

____

If we put flies on ice for an increasing amount of time (1 minute, 5 minutes, 10 minutes) then I expect that the time it takes flies to recover to (circle one, then explain):

INCREASE   STAY THE SAME   DECREASE

Because______________________________________

____

____

____

ESTABLISHING OUR PROCEDURE

How will we test our scientific question?

Use the box below to explain the procedure you will use to test your hypothesis.

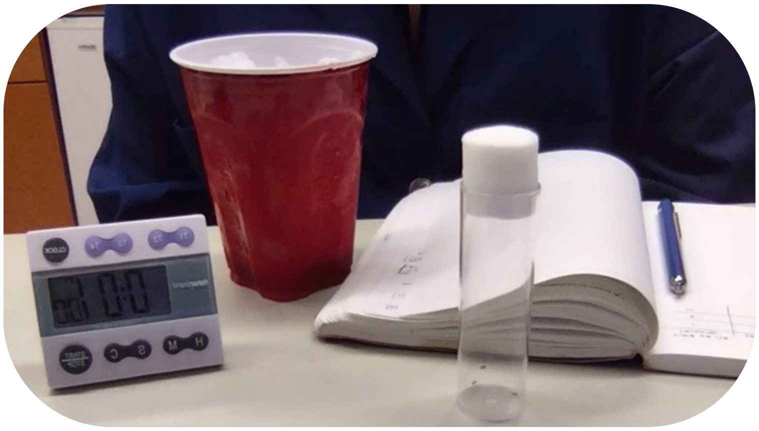

Materials that we will use in this experiment

WHAT ARE THE VARIABLES IN OUR EXPERIMENT?

In an experiment, **variables** are elements, features, or factors that can "vary" or change. Below list independent and dependent variables for our "flies on ice" experiment. Also include a list of controlled variables.

Independent variable	*Dependent variable*	*Controlled variables*
The element, feature, or factor that the scientist changes, manipulates or adapts in an experiment.	The element, feature, or factor that the scientist measures in an experiment.	Any element, feature, or factor that does not change and is held constant in an experiment.
________________<br>________________<br>________________<br>________________<br>________________<br>________________<br>________________<br>________________<br>________________	________________<br>________________<br>________________<br>________________<br>________________<br>________________<br>________________<br>________________<br>________________	________________<br>________________<br>________________<br>________________<br>________________<br>________________<br>________________<br>________________<br>________________

DATA COLLECTION

Doing our experiment

Below record the recovery time of your flies after 1, 5, and 10 minutes on ice. Use additional lines to record replicates and/or your class-wide average.

Recovery time

The time in takes one half of the number of flies in the vial to flip themselves over

1 minute on ice

Recovery time (in seconds)

____

____

5 minutes on ice

Recovery time (in seconds)

____

____

10 minutes on ice

Recovery time (in seconds)

____

____

1 minute on ice

Class average recovery time (in seconds)

____

5 minutes on ice

Class average recovery time (in seconds)

____

10 minutes on ice

Class average recovery time (in seconds)

____

Use this space to record any additional observations you make

____

____

GRAPHING OUR RESULTS

Communicating our data

Graph title: ______________________________

Y axis title (dependent variable)

X axis title (independent variable)

Describe the relationship between the variables in a few sentences

____

____

____

INVESTIGATION DAY 3

Analyzing our results

For each graph, describe the relationship between the variables ***time on ice*** *and* ***recovery time****.*

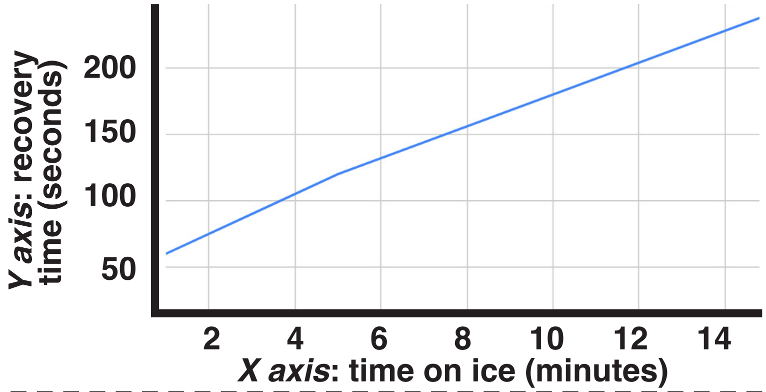

Graph A

____

____

____

____

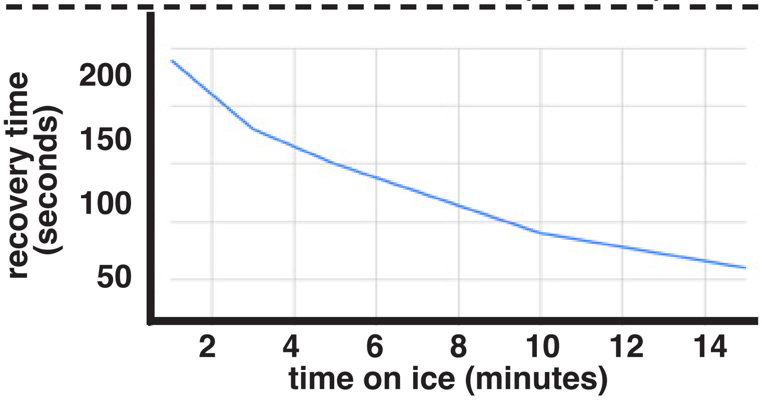

Graph B

____

____

____

____

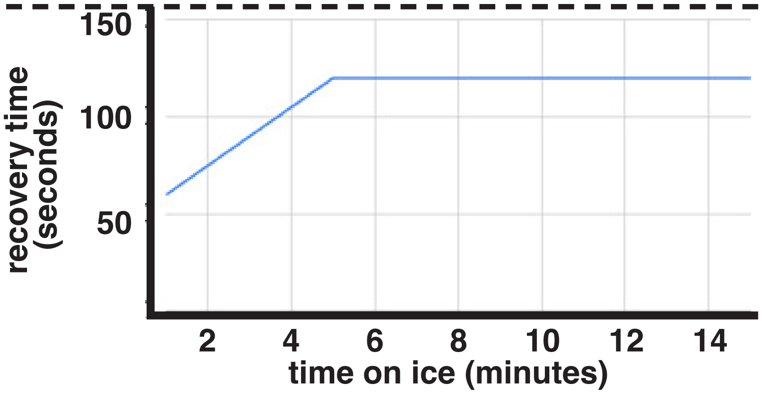

Graph C

____

____

____

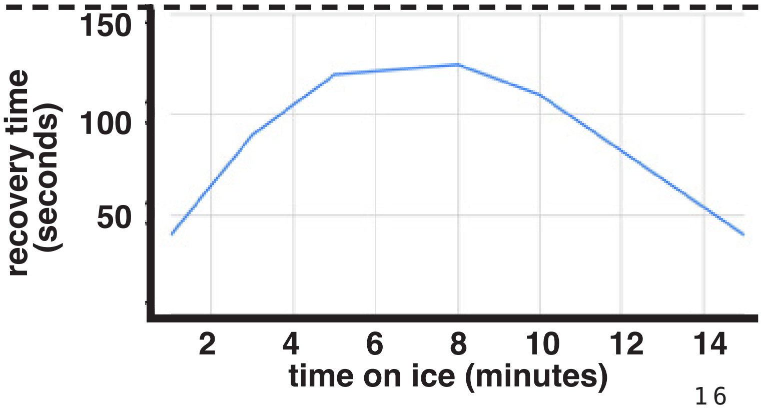

Graph D

____

____

____

Based on your results, how does exposure to ice for 1, 5, and 10 minutes affect the flies' recovery time?

Claim

(a 1 sentence answer to the question)

____

____

____

____

Evidence

(at least 2 relevant observations you made that support your claim)

____

____

____

____

Reasoning

(explain how your evidence supports your claim)

____

____

____

____

____

Do your results support your original hypothesis?

YES NO

THE BIOLOGY OF FLIES ON ICE

What happens in the fly nervous system when we put them on ice?

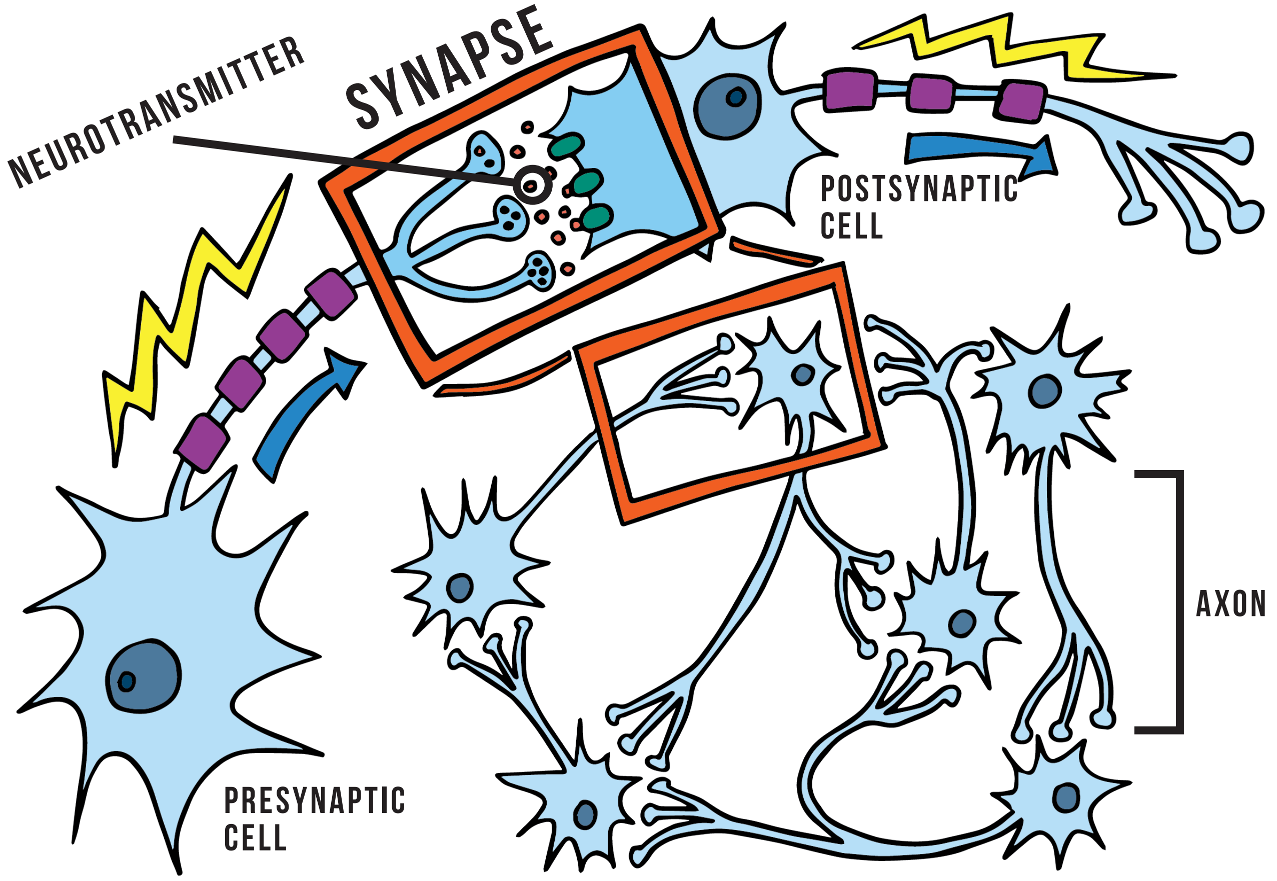

A close-up view of the electrical and chemical signals that occur between neurons at the synapse. Illustration by Kelly Sullivan.

Neurons are the wires of the nervous system, and they connect together to form signaling networks. Electrical signals travel down the **axon** and cause vesicles containing **neurotransmitters** to be released from the axon into the **synapse**. This binding of neurotransmitters to various cells, such as a muscle cell, will elicit a reaction, such as a muscle contraction. If neurotransmitters bind to another neuron, like in the illustration above, that **postsynaptic** neuron will react (or not react) to the signal. The neurotransmitters that are left over in the synaptic cleft are taken up by the **presynaptic** cell to be used again.

This process is very sensitive to temperature, so if the neurons and muscles get too cold, the activity of proteins, such as **enzymes**, falters and they stop working. Once the flies are taken off ice and the body temperature warms up, protein activity resumes, allowing the presynaptic cell to reabsorb the neurotransmitters so that it can "fire" again.

NOTES

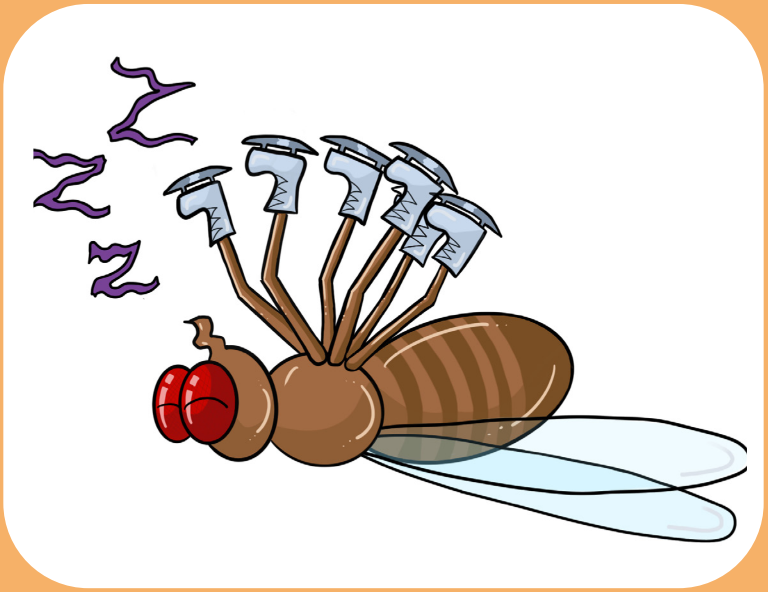

Supplement: Extended Data 2 — Flies on Ice student workbook. Download Extended Data 2, ZIP file. [file enu-eN-NWR-0263-22-s02.zip › Supplemental 1 - Flies on Ice Student Workbook.pdf]
