# Supplementary material for: DrosoPHILA: A Partnership between Scientists and Teachers That Begins in the Lab and Continues into City Schools
Source: eNeuro. 2023 Feb 13;10(2):ENEURO.0263-22.2022. doi: 10.1523/ENEURO.0263-22.2022 (PMC9927510; doi:10.1523/ENEURO.0263-22.2022)
Supplement: Extended Data 3 — Lesson plans for Roundabout We Go! Download Extended Data 3, ZIP file. [file enu-eN-NWR-0263-22-s06.zip › Supplemental file 4 - Roundabout teacher notes.pdf]

And a Roundabout We Go

Using *Drosophila* as a model organism to study the effects of mutations on gene expression

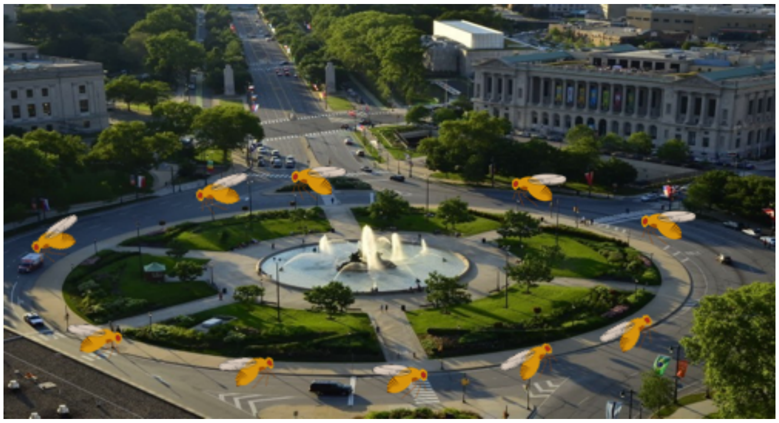

Objectives:

(1) SWBAT design an experiment, follow a protocol, collect and analyze data IOT investigate a scientific question

(2) SWBAT describe how genetic mutations (nucleotide sequence) relate to changes at the molecular and organismal levels in terms of fruit fly nervous system development and larval behavior.

Table of Contents:

I. General Overview and Suggested Timeline…………………………. p.2

II. Connections to the Biology Curriculum…………………………….. p.3

III. Background Information for teachers ……………………………… p.4

IV. Specific Suggestions for Implementation…………………………… p.7

V. Standards Alignment……………………………………………….... p.12

VI. References………………………………………………………….... p.14

I. General Overview and Suggested Timeline

This activity allows students to directly compare behavioral phenotypes and consider the genetic and molecular causes. Although observation of larval fruit fly behavior could be used as an hook to engage students in study of genetics, these activities would work best to contextualize existing understanding of basic genetics including inheritance patterns and the mechanism by which genotype determines phenotype. Students should already be familiar with the concept of genes, alleles, Mendelian patterns of inheritance, Punnett squares, and gene expression through transcription and translation. Familiarity with mutations as changes in nucleotide sequence is helpful.

Day 1 - Show videos of the child with mirror movement disorder and the hopping mouse; Discuss the benefits of model organisms; Discussion on the genetics of making flies with abnormalities; Review of DNA transcription and translation; Students view embryo slides under compound light microscopes and describe phenotypes.

Day 2 - Students conduct an experiment to analyze larval crawling tracks. Students will determine which tracks belong to the wild type vs the mutant, and also make predictions to which embryo nerve cord best corresponds to the larval crawling pattern. Students will practice gathering data and developing questions based on observations.

Day 3 - Explain how mutations with the robo receptor can affect nervous system development and subsequently larval crawling patterns

***One or two days prior to beginning this unit, administer the provided pre-assessment. Students should not write their name on the survey. Rather, they should use their 7 or 8-digit school district ID number that they will use to identify themselves again on the post-assessment, to be administered either at the end of class on Day 3 (if there is time) or the following day.*

II. Connections to the Biology Curriculum

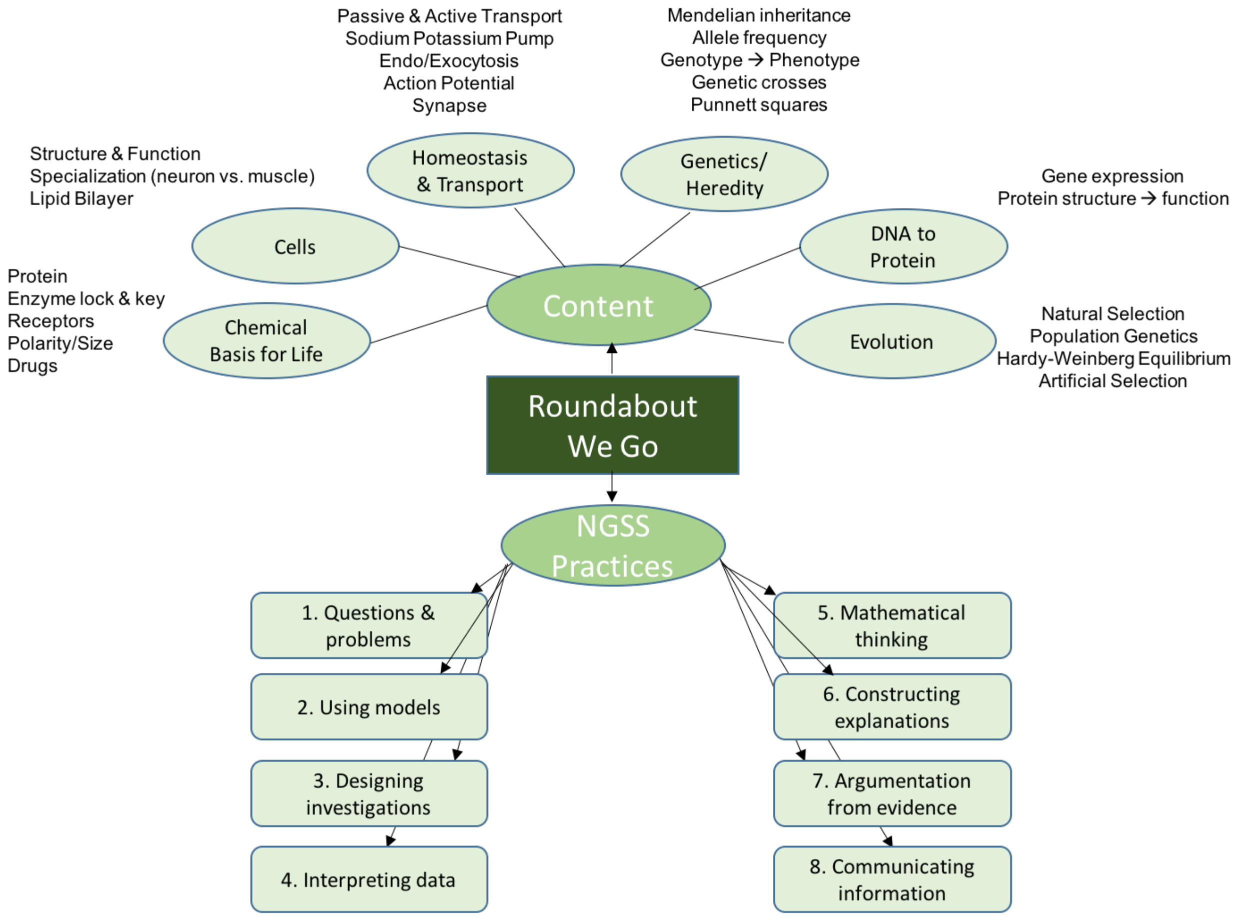

III. Background Information for teachers

Drosophila melanogaster has four distinct phases of its life cycle: embryo, larva, pupa and adult. It takes about 10 days for this process to occur at 25°C. Approximately one day after fertilization, the *Drosophila* embryos develop and hatch into a worm-like larvae. The larvae continue to grow and proceed through multiple stages known as "instars", with intervening "molts". Larval development lasts about five days. Larvae then go through an immobile pupal stage, lasting about four days, before emerging as an adult fly. In this unit, students will be working with embryos as well as 3rd instar larvae (the last larval stage before becoming a pupa).

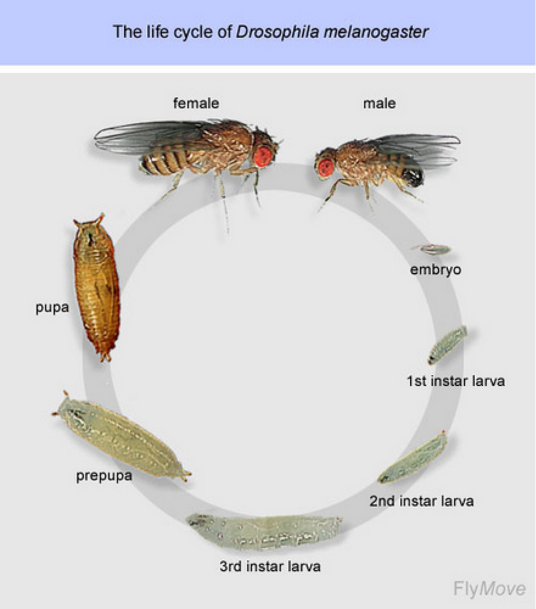

Table 8.2 Larval stages of *Drosophila*

Time after fertilization		
**Hours**	**Days**	**Developmental event (at 25°C)**
24	1	Hatching from egg; first larval instar begins
49	2	First molt; second instar begins
72	3	Second molt; third instar begins
120	5	Puparium formation; puparium white
122	5.1	Puparium fully colored
124	5.2	"Prepupal" molt
132	5.5	Pupation; cephalic complex, wings, legs everted
169	7	Eye pigmentation begins
189	7.9	Bristle pigmentation begins
216	9	Adult ready to emerge from pupa case

Source: After Doane, 1967.

https://www.flickr.com/photos/11304375@N07/2993342324
http://web.as.uky.edu/Biology/faculty/cooper/Population%20dynamics%20examples%20with%20fruit%20flies/08Drosophila.pdf

Humans and flies are examples of bilaterally symmetric organisms, meaning that their bodies can be divided into matching halves by drawing a line down the center. This imaginary line is known as the "midline" and is represented by the blue dashed line in the figure to the right. The ability of such organisms to properly coordinate movement between the left and right sides of the body relies on the normal development of neuronal connections during embryogenesis.

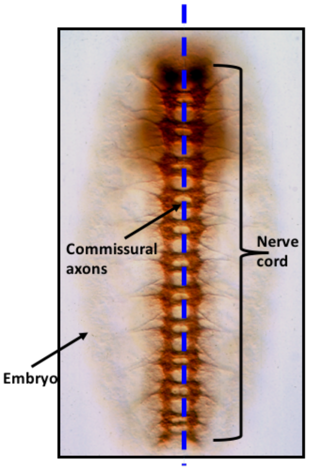

The developing nervous system contains neurons that send their axons across the midline (known as commissural axons) and those that do not (longitudinal axons). Many "ligands", or cues, that either attract or repel these growing axons, are produced by cells at the midline. Growing commissural axons (those that cross the midline) initially respond to attractive signals emanating from the midline, while ignoring repulsive cues. Once the commissural axons cross

the midline, repulsion dominates, allowing axons to exit away from the midline while preventing them from re-entering (Evans & Bashaw, 2010). These processes are mediated by ligand/receptor systems that are conserved (have conserved protein sequences and protein function) among vertebrates and invertebrate organisms. Ultimately, these attractive and repulsive systems allow the nerve cord to develop properly during embryogenesis.

These ligand/receptor systems include:

- Slit ligand and Roundabout (Robo) receptor (which promote axon *repulsion* away from the midline)
- Netrin ligand and DCC family of receptors (which promote axon *attraction* towards the midline; note, in *Drosophila*, the DCC receptor is known as Frazzled or Fra, for short)

The Repulsive Guidance System

The Slit ligand (produced by midline cells) binds to the Robo receptor (being expressed on the tips of the axon) and causes axons to turn away from the source of the ligand. In the case of commissural axons, once across the midline, it facilitates their exit and prevents them from abnormally crossing back to the other side (Evans & Bashaw, 2010). You might imagine then in a *robo* loss-of-function mutant, repulsion is absent and axons are now free to cross and recross the midline, creating an embryonic nerve cord that has thicker commissures and thinner longitudinal tracts (see the figure to the right; compare to nerve cord on previous page).

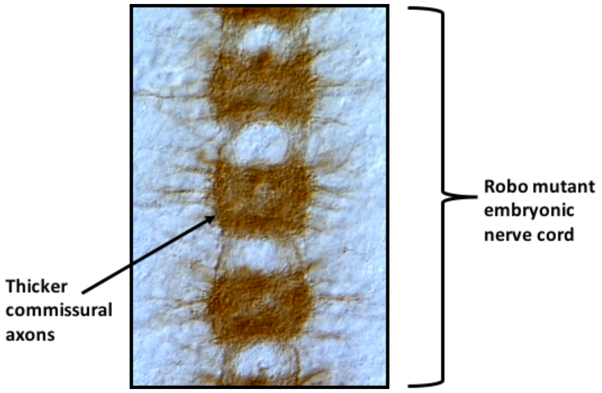

The protein Commissureless (Comm) promotes midline crossing by decreasing surface levels of the Robo receptor (Neuhaus-Follini & Bashaw, 2015). For commissural axons, Comm is initially turned on thereby sorting the Robo receptor to endosomes and lysosomes (before the receptor reaches the cell surface) where it is degraded (Keleman et al, 2002). In other words, Comm activity basically turns off the repulsive system, allowing axons to respond to the attractive signals at the midline. Once the axon crosses to the other side, Comm is turned off, allowing Robo receptors levels to increase thereby re-establishing repulsion from the midline (see the bottom left figure). *comm* loss-of-function mutants do not express the Comm protein,and this results in increased levels of Robo on axons before midline crossing. This excess Robo causes commissural axons to respond prematurely to the repulsive Slit signal, preventing all axons from approaching the midline (see the middle figure on the next page).

Mechanism of Comm Action | *comm* lof mutant | *slit* lof mutant

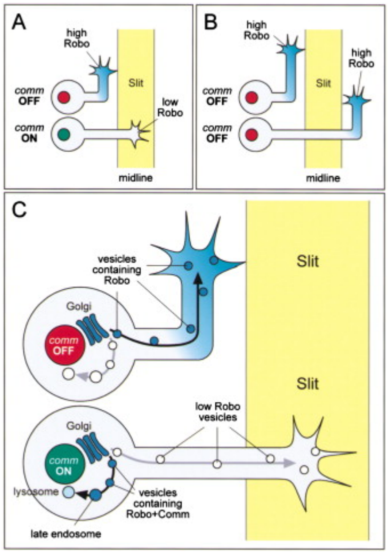

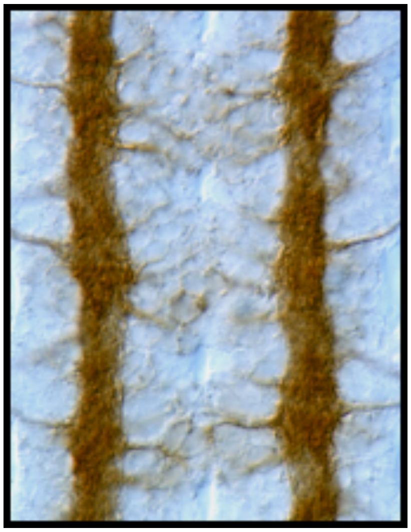

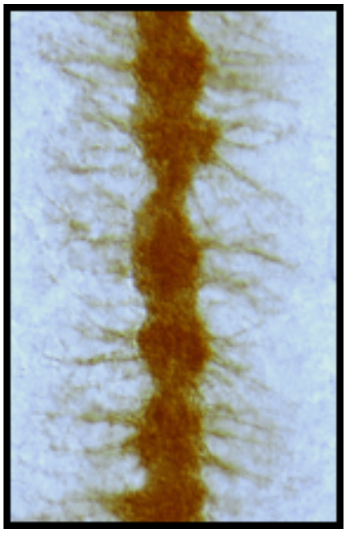

In *slit* loss-of-function (lof) mutants, axons respond to midline-generated Netrin ligand, enter the midline, but fail to exit due to a lack of repulsion (see above image on the right). The Fra receptor is also involved in the regulation of Comm expression, independent of its ligand Netrin. The exact mechanism of how this works is currently one of the topics of research at the Bashaw Lab at the University of Pennsylvania!

If the *Drosophila* embryo survives, it will move onto the larval stage where it develops mouth hooks and the ability to crawl. Wild type larvae typically exhibit straight line crawling patterns interrupted at intervals by pause turns that halt crawling and redirect the trajectory of movement (Berni, 2015). The forward crawls of *robo* mutant larvae, however, follow a circular path without sharp redirections generated by pause turns, limiting their movement in any one direction. Wild type larvae crawling tracks vs *robo* mutant larvae crawling tracks, respectively:

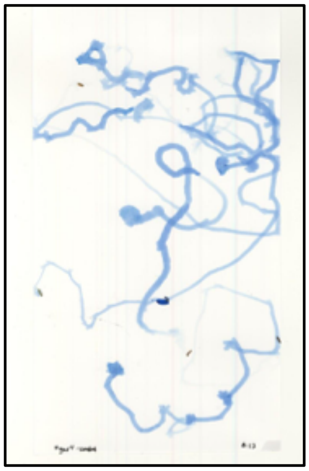

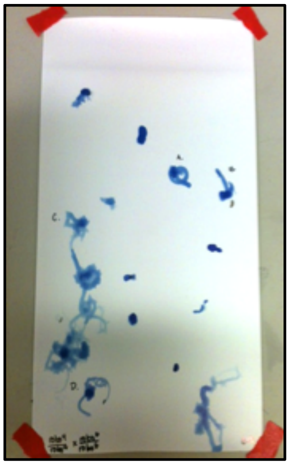

IV. Specific Suggestions for Implementation

<table>
<tr><th colspan="2">Day 1 Agenda</th></tr>
<tr>
<td>
Student workbook pages 1 - 9.

Slide deck slides 1 - 22.

Materials needed per group of 4 students:

1-2 compound light microscopes
A set of prepared embryo and life cycle slides or images of prepared embryos

Optional supplementary materials

Plastic page protectors
Dry erase marker & eraser

Key Vocabulary:

Model organism
Homozygous
Heterozygous
Punnett square
Mutation
Genotype
Phenotype
Embryo

</td>
<td>

Hook: Show students the video of a child with mirror movement disorder and of the hopping mouse and ask students to make some general observations about their behaviors (Page 4)
Review DNA transcription and translation, neurons, and mutations. (Page 4 - 5)
Use video of mice movement between wild type and those with a mutation to lead into a discussion about the importance of model organisms in research (Page

Do you think it is ethical to manipulate the cellular processes of the child to learn more about his condition? How else could we study development in humans, other than manipulating actual humans? What are advantages and disadvantages of studying a similar phenomenon in mice? What about flies?

Show examples of similarities between humans and flies (particularly in their bilateral symmetry and nerve/spinal cord structure)
Briefly explain to students how flies with known mutations can be created
Give students a chance to predict the offspring of a cross between two flies heterozygous for a nerve cord mutation.
In groups of 4, students use microscopes to observe prepared embryo slides, and a life cycle slide. Students will record their observations on the student handout.
Wrap-up: Review which slides corresponded to which type of nerve cord mutation and whether the mutations are dominant or recessive

Teacher reference: “A = Comm mutants”; “B = Slit mutants”; “C = Robo mutants”
Optional: gather class data on wt:mutant ratio. Discuss how outcome compares to prediction.

</td>
</tr>
</table>

<table>
<tr><th colspan="2">Day 2 Agenda</th></tr>
<tr>
<td>
Student workbook pages 10 - 14.

Slide deck slides 27 - 41.

Materials needed per group of 4 students:
● ~10 mL of 15% sucrose solution
● ~1 drop of at least 3 different colors of food coloring
● 1 vial of larvae labeled “X”
● 1 vial of larvae labeled “O”
● 1 Empty petri dish
● Water
● 1 small paintbrush
● Photo paper
● 1 paper towel
● Rubber band

Key Vocabulary:
● Mutation
● Phenotype
● Midline
● Claim
● Evidence
● Reasoning
</td>
<td>
● Brief review of Day 1’s lesson by showing and discussing photos students took through the microscope (or images provided).
● Ask students what the embryos would turn into if left another day. Use page 10 to review the behaviors of the embryo and larval stages of the fruit fly life cycle.
● Tell students that today they’ll be looking at the larvae that they’d see 3 days from yesterday. Encourage students to ask questions especially about how the differences observed might relate to larval function such as movement and survival. Students complete a hypothesis on how the robo mutation at the embryo stage would affect the larval stage. (Page 10)
● Demonstrate how to collect larvae from a vial (see below for detailed notes on this process!) and how to stain them with food coloring.
● Distribute supplies. NOTE: You will need to put one drop of at least 3 different colors of food coloring onto the lid of the petri dish before class begins! See the image below for an example of how it should look:
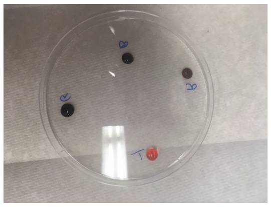
● NOTE: Each group of students will receive two vials of larvae, marked with either an “O” or an “X”. Each pair within the group will work with one of the vials. Each pair of students should collect and stain a minimum of 3 different larvae. Students record their data on page 12.
    ○ Based on the larval crawling patterns, students figure out which vials of larvae might be classified as wild type and which might be classified as mutant.
        ■ Teacher only: “X” are wildtype; “O” are mutant.
● Students make a prediction as to which type of mutant the larvae might be (robo, comm or slit). Then,
</td>
</tr>
</table>

	they discuss why, in the wild, such mutants might not make it to the adult stage. Students complete a claim with evidence and reasoning on pages 13 and 14.<br>● CLEAN-UP:<br>○ Students will need to keep their larvae, as they will be used again (either with another class or for Day 4's Fly art activity). See below for detailed notes on how to "save the larvae"!<br>○ Rinse the paint brushes and petri dishes with water and pat them dry.<br>● By the end of class, students should be done with Day 2 of their packets. If they don't finish during class, you can choose to assign it for homework or give them time to work on it at the beginning of Day 3.<br>● Wrap-up.

<u>How to collect and stain fly larvae:</u>

1. Add a small amount (approximately 10 mL or until it reaches halfway up the vial) of sucrose solution. Wait until you see about 5-6 larvae float up to the surface:

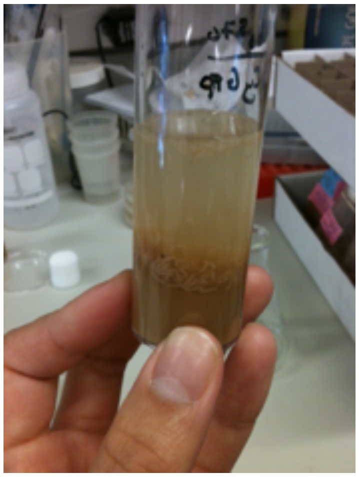
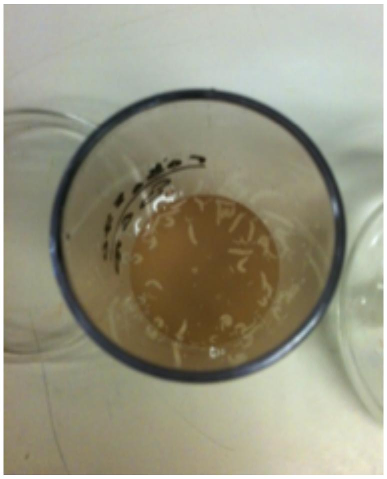

2. Open up the petri dish. On the cover, use a pipette to add one drop of food coloring.
3. Use the paintbrush pick out 3-4 larvae directly out of the vial and place them on the shinier side of the photo paper.
4. Dip the paintbrush into the drop of food coloring and gently dab each of the larvae with the dye. Dab your larvae with food coloring as needed (if the larvae dries out or the

color starts to fade). Use the smallest amount of food coloring possible otherwise the larvae get stuck.

<u>How to "save the larvae" to be used again for another class OR for the fly art activity on Day 4:</u>

1. Use a rubber band to secure a paper towel across the top of your vial.
2. Pour out as much liquid as possible (into a waste cup or the sink).

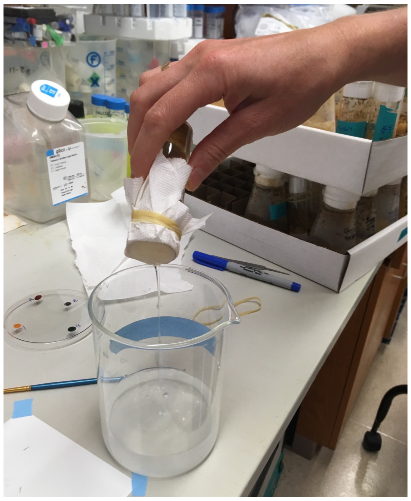

3. Poke holes in the paper towel if it becomes clogged.
4. Throw the paper towel in the trash.

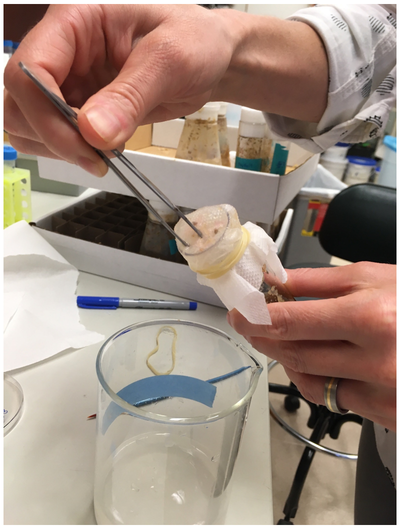

**Day 3 Agenda**	
Student workbook pages 14 - 19.<br><br>Slide deck slides 42 - 53.<br><br>Key Vocabulary:<br>● Ligand<br>● Receptor<br>● Gene<br>● Transcription<br>● Translation<br>● Qualitative<br>● Quantitative	● Review the results from the experiment on Day 2<br>● Explore qualitative versus quantitative methods of collecting data (Page 14 – 15)<br>● Using page 16, Discuss the molecular basis of how changes in the expression of the Robo receptor can lead to changes in the embryo nerve cord and subsequently larval crawling behavior patterns (Page 17)<br>○ Show the video of a neuron responding to attractive and repulsive cues that cause the axon to grow and shrink, respectively<br>● Make connections between nervous system development and the levels of organization (Page 18 - 19)

	• Review the central dogma and how DNA codes for protein<br>• To wrap-up:<br>○ Review translation of the Robo1 gene<br>○ Review how mutations in the Robo1 gene receptor can lead to the mutant crawling patterns observed on Day 2

V. Standards Alignment

I. Next Generation Science Standards

HS. Structure and Function

Students who demonstrate an understanding can:

HS-LS1-1. Construct an explanation based on evidence for how the structure of DNA determines the structure of proteins which carry out the essential functions of life through systems of specialized cells

HS. Inheritance and Variation of Traits

Students who demonstrate an understanding can:

HS-LS3-1. Ask questions to clarify relationships about the role of DNA and chromosomes in coding the instructions for characteristic traits passed from parents to offspring.

HS-LS3-2. Make and defend a claim based on evidence that inheritable genetic variations may result from: (1) new genetic combinations through meiosis, (2) viable errors occurring during replication and/or (3) mutations caused by environmental factors

HS. Natural Selection and Evolution

Students who demonstrate understanding can:

HS-LS4-4. Construct an explanation based on evidence for how natural selection leads to adaptation of populations.

II. Pennsylvania State Science Curriculum

Standard 3.1.B.A9

- Identify questions and concepts that guide scientific investigations.
- Know that both direct and indirect observations are used by scientists to study the natural world and universe.
- Evaluate experimental information for relevance and adherence to science processes.
- Interpret results of experimental research to predict new information, propose additional investigable questions, or advance a solution.

Standard 3.1.B.B1

- Explain that the information passed from parents to offspring is transmitted by means of genes which are coded in DNA molecules.
- Explain how mutations can alter genetic information and the possible consequences on resultant cells.

Standard 3.1.B.B5

- Describe how Mendel's laws of segregation and independent assortment can be observed through patterns of inheritance.

III. Pennsylvania Biology Keystone Assessment Anchors

BIO.A.1: Basic Biological Principles

BIO.A.1.2: Describe relationships between structure and function at biological levels of organization.

BIO.B.2: Genetics

BIO.B.2.1: Compare Mendelian and non-Mendelian patterns of inheritance.

BIO.B.2.2: Explain the process of protein synthesis (i.e., transcription, translation, and protein modification).

BIO.B.2.3: Explain how genetic information is expressed

VI. References and recommended reading

Berni, J. Genetic dissection of a regionally differentiated network for exploratory behavior in *Drosophila* larvae. Curr Bio (2015). 25: 1319-1326.

Depienne *et al.* RAD51 Haploinsufficiency Causes Congenital Mirror Movements in Humans. Am J Hum Genet (2012). 90(2):301-307

Evans, T., Bashaw, G. Axon guidance at the midline: of mice and flies. Curr Opin Neurobiol (2010). 20(1): 79-85.

Held, Lewis I. Jr. Deep Homology?: Uncanny Similarities of Humans and Flies Uncovered by Evo-Devo (2017). pgs.46- 53 Cambridge University Press.

Keleman, K., Rajagopalan, S., Cleppien, D., Teis, D., Paiha, K., Huber, L., Technau, G., Dickson, B. Comm sorts robo to control axon guidance at the *Drosophila* midline. Cell (2002). 100 (4): 415-427.

Neuhaus-Follini, A., Bashaw, G.J. Crossing the embryonic midline: molecular mechanisms regulating axon responsiveness at an intermediate target. WIREs Dev Biol (2015).

Roze, Emmanuel Flamand. Mirror movement disorder: A study of congenital mirror movement in a French family. (2012) Note: This is a great example of how population genetics and heredity can be studied with pedigrees. https://www.youtube.com/watch?v=zL7395UNXFY

Tyler, Mary S. Development of the fruit fly *Drosophila melanogaster* (2000). Developmental Biology, a guide for experimental study, Second Edition. Access chapter online: http://web.as.uky.edu/Biology/faculty/cooper/Population%20dynamics%20examples%20with%20fruit%20flies/08Drosophila.pdf
