# Supplementary material for: DrosoPHILA: A Partnership between Scientists and Teachers That Begins in the Lab and Continues into City Schools
Source: eNeuro. 2023 Feb 13;10(2):ENEURO.0263-22.2022. doi: 10.1523/ENEURO.0263-22.2022 (PMC9927510; doi:10.1523/ENEURO.0263-22.2022)

SPRING 2022

ROUNDABOUT WE GO!

A DROSOPHILA INVESTIGATION

Name ______________________ Student ID ______________________

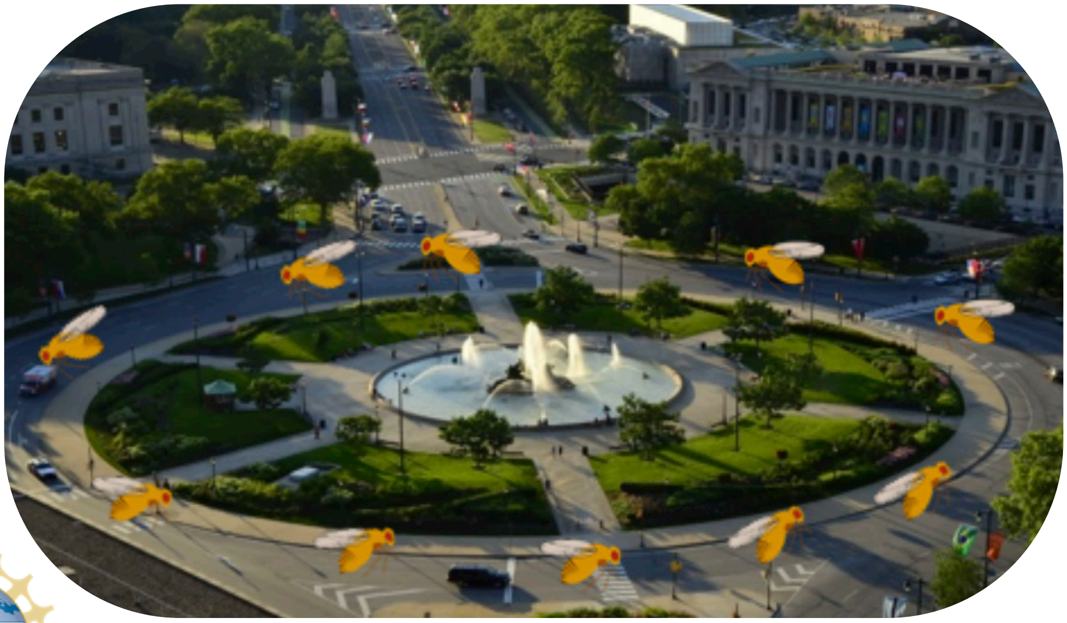

IOS 1853719

TABLE OF CONTENTS

3 WELCOME

Meet the scientists and educators behind DrosoPHILA

4 MOVEMENT AND THE NERVOUS SYSTEM

Learn how animals move and how neural circuit formation makes that movement possible.

7 INVESTIGATION, DAY 1

Observe embryos with mutations that affect neural circuit formation.

10 INVESTIGATION, DAY 2

Perform an experiment to test the effect of genes on behavior in fly larvae.

14 INVESTIGATION, DAY 3

Analyze your data and learn abotu the relationship between genes and behavior.

DrosoPHILA
IOS 1853719

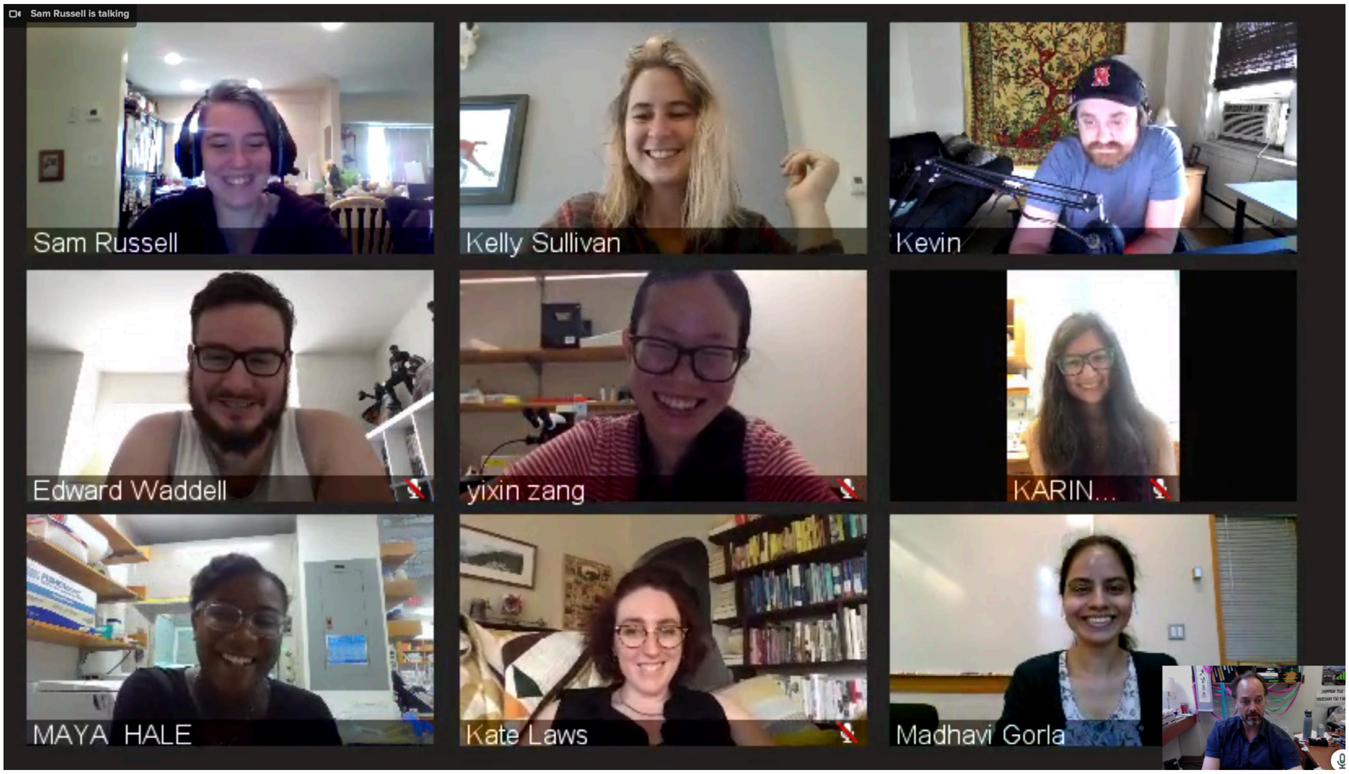

Members of the Bashaw lab gather virtually in August 2020

WHO WORKS IN A LAB?

PRINCIPAL INVESTIGATOR Manages all projects in the lab, applies for funding, and sometimes teaches classes. Has a Ph.D. and often postdoctoral research experience.

POSTDOCTORAL RESEARCHER Develops an independent research program as an apprentice to the principal investigator and sometimes teaches classes. Has a Ph.D.

GRADUATE STUDENT Works on an independent research project under the supervision of the principal investigator while taking classes. Can be pursing a Ph.D. or a Master's degree. Has at least a Bachelor's degree. Master's programs are usually 1-2 years long. Ph.D. programs can take 5 years. A Master's degree is not required to pursue a Ph.D.

RESEARCH ASSISTANT Supports projects in the laboratory and sometimes develops their own project. Research assistant positions can be pursued with a Bachelor's degree. People who hold an Associate's, Bachelor's, Master's degree, or Ph.D. can also work professionally as research assistants, specialists, or technicians.

LAB MANAGER A professional position that typically requires at least a Bachelor's degree. Lab managers are responsible for ordering materials, maintaining equipment and space, and scheduling meetings. They are usually familiar wtih the research in the lab and may assist with research.

MOVEMENT AND THE NERVOUS SYSTEM

Observe the child working on the puzzle. What is unusual about their behavior? Record three observations in the box below

____

____

____

WHAT IS THE RELATIONSHIP BETWEEN GENES AND BEHAVIOR?

Molecules organize to form **DNA**. DNA encodes specific genes, which are transcribed into **mRNA**. mRNA is translated into **proteins** by **tRNA** at the **ribosome**.

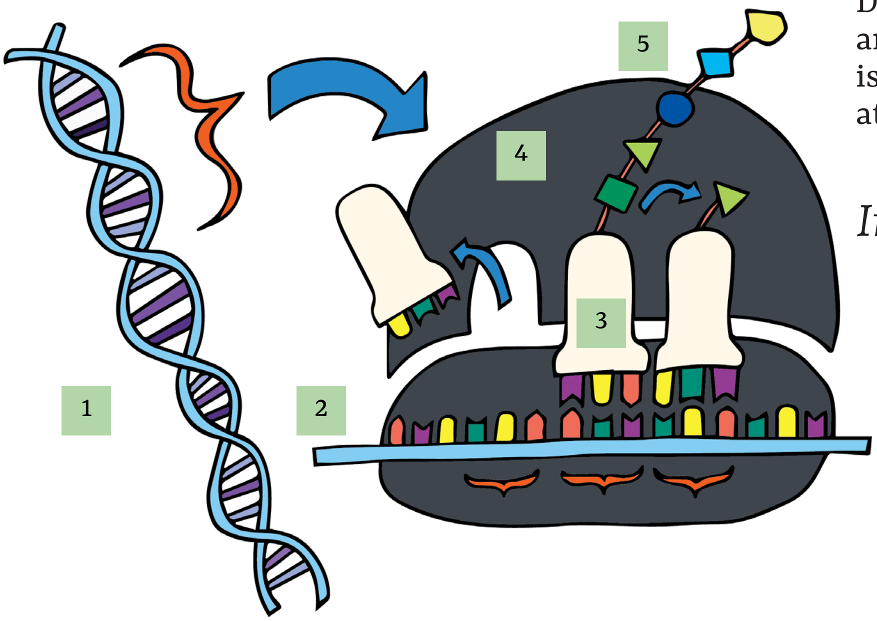

In the box below, label the parts of the illustration.

1 ______________________

2 ______________________

3 ______________________

4 ______________________

5 ______________________

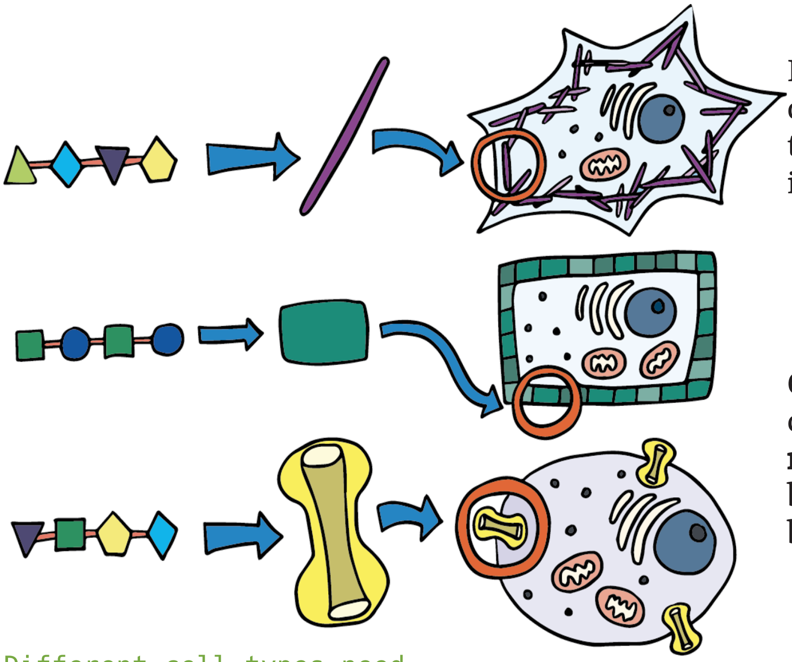

Different **cells** in an organism express different genes. The proteins encoded by the genes expressed in a cell determine its shape and function.

Cells, including **neurons**, organize to form complex **tissues** and organs, including **neural circuits**. Organisms are able to behave (for example, they can move) because of the nervous system.

Different cell types need different types of proteins.

Use the box below to record the types of signals neurons send to their partners.

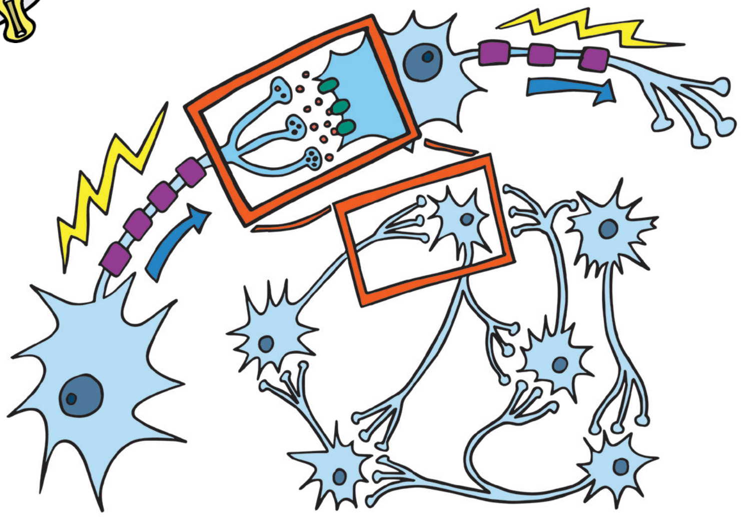

Neurons organize into circuits

How do you think a change in DNA sequence could affect an animal's movement?

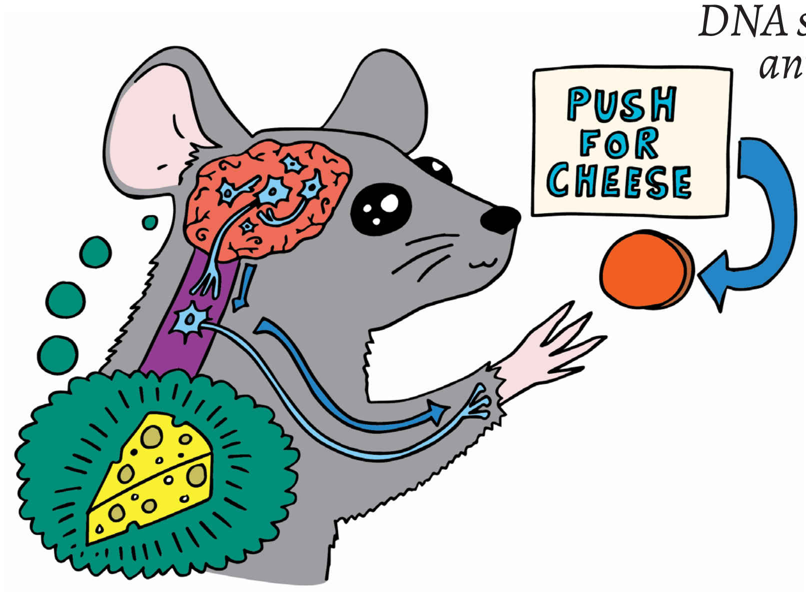

Observe the mouse walking. This mouse has a mutation in a gene that is important for neural circuit formation. What do you observe about its behavior? How is this similar to the behavior of the child in the previous video?

Should we study changes in neural circuitry in humans or in mice? What makes you say that?

DROSOPHILA AS A MODEL ORGANISM

What does it mean to be a "model organism"?

What behaviors can we observe in both flies and humans?

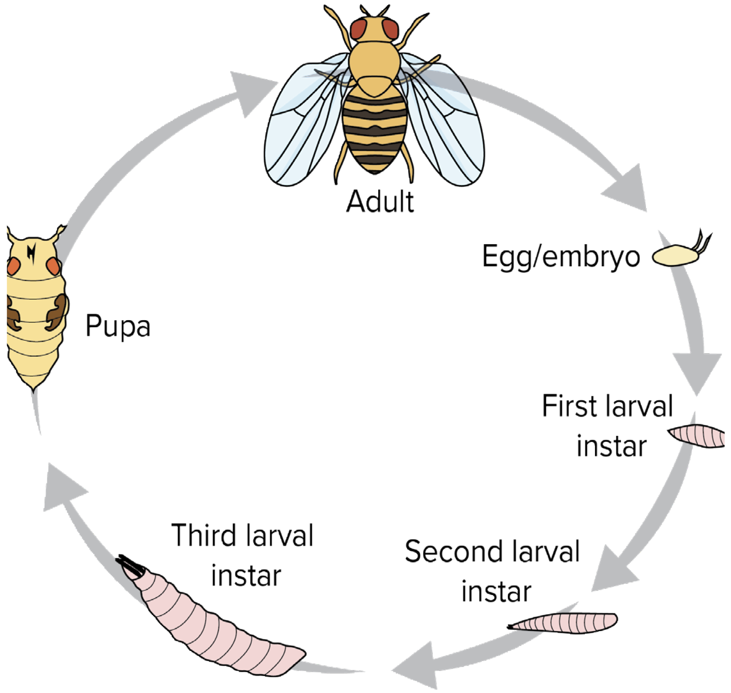

Circle the stage of the life cycle where neural circuits are first formed.

INVESTIGATION DAY 1

Which genes are important for nervous system formation?

As you saw prevoiusly, while all cells in an organism have the same **DNA**, not every cell will express the same genes. These differences in **gene expression** are what make cells look and behave differently from each other. We are interested in understanding how the nervous system develops, especially which genes encode proteins that are used to form neural circuits.

To understand which genes are important for nervous system development, scientists disrupt gene function and see if it also disrupts formation of the nervous system. These disruptions in gene function are caused by **mutations**, changes in the DNA sequence.

The mutations that we are studying are **homozygous lethal**, meaning that **homozygous** organisms die before they reach adulthood. We can still study homozygous organisms in very early development. We do this by setting up cages (like the one in the photograph to the right) to collect embryos from **heterozygous** parents. The adult flies lay eggs on apple juice agar plates, and if the eggs are fertilized, they become embryos.

After collecting the embryos off of the plate, we stained them to make the nerve cord visible. This will allow you to see the **morphology**, or structure, of the nerve cord and observe changes in the morphology of the nerve cord in mutant embryos. You can see an example of wild-type morphology in the image to the left.

Finally, we placed the stained embryos on a slide with a coverslip. These are the slides that you will now observe under the microscope. **Use the following page to record your observations** and determine whether or not the mutated gene is important for nervous system development.

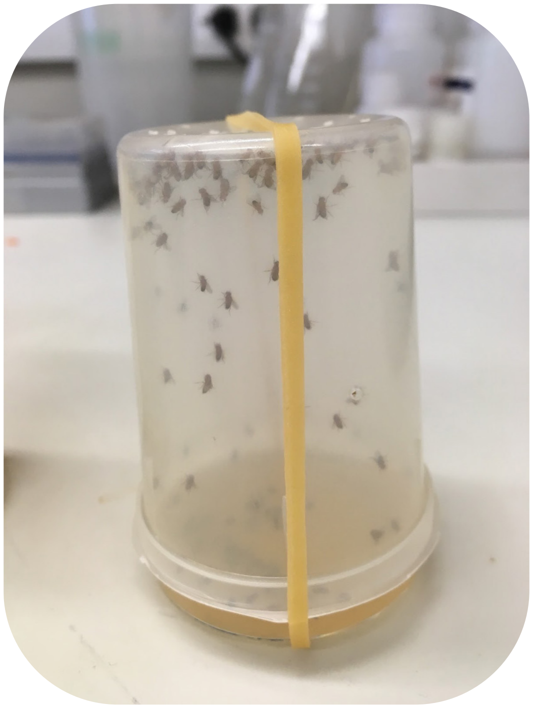

Scientists collect embryos for analysis using cages like this one. Adult flies lay their eggs on plates made with apple juice or another sugary substance. If the eggs are fertilized, they develop as embryos.

Fill out the Punnett square below to demonstrate what offpsring can be produced when two ***heterozygous*** *parents mate. Use "R" to represent the dominant, wild-type allele and "r" to represent the recessive, mutant allele.*

You have recorded the ***genotypes*** *of the offspring. What is the ratio of wild-type to mutant* ***phenotypes*** *among offpsring?*

____

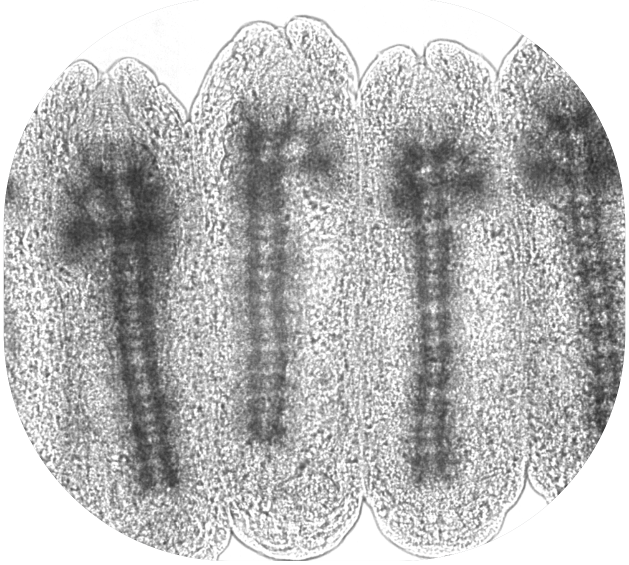

A group of wild-type embryos observed under a microscope

OBSERVATIONS

Look at each of the three slides. Draw an image of the mutant phenotype in the corresponding box. Record the ratio of embryos with mutant and wild-type phenotypes on that slide in the box next to your drawing.

A

wild-type	*mutant*

B

wild-type	*mutant*

C

wild-type	*mutant*

INVESTIGATION DAY 2

What are the consequences of disrupted circuit development?

Refer back to the life cycle on page 7. What types of behavior could you observe in embryos?

Refer back to the life cycle on page 7. What types of behavior could you observe in larvae?

Scientific question: how might the robo mutation, which you observed at the embryonic stage, affect behavior in the larval stage of fly development?

ESTABLISHING OUR PROCEDURE

How will we test our scientific question?

Before we begin our experiment, use the space below to record the purpose of each material in the experiment. Then, list the independent, dependent, and control variable in the boxes at the bottom of the page.

Materials	*use in this experiment*
Genotype X and Genotype O larvae	______________________
Food coloring	______________________
Paintbrush	______________________
Shiny paper	______________________
Tape	______________________

Independent variable	*Dependent variable*	*Control variables*
______________	______________	______________
______________	______________	______________
______________	______________	______________
______________	______________	______________
______________	______________	______________

DATA COLLECTION

Use the space provided to draw larval tracks for each genotype. Record your observations about the tracks for each genotype in the green box to the right.

Sketch (Genotype X)

Observations (Genotype X)

Sketch (Genotype O)

Observations (Genotype O)

ANALYSIS OF RESULTS

Based on your results, which vial contained wild-type larvae?

Claim (a 1 sentence answer to the question)	
Evidence (at least 2 relevant observations you made that support your claim)	
Reasoning (explain how your evidence supports your claim)	

Without knowing which vial contained mutant larvae, you conducted this study "blind". What are some reasons that scientists may decide to "blind" their experiments?

INVESTIGATION DAY 3

Extending our analysis

Was the evidence that you used to support your argument qualitatitve or quantitative? Explain.

Qualitative data is information describing something as opposed to counting it. How would you describe the larval tracks?

___

___

___

___

Quantitative data is information relating to the number of something as opposed to a description of it. How might you quantify, or give a number to, the larval tracks?

___

___

___

___

___

*Use the space to the right to make a table to **quantify** the data you collected yesterday. Make sure to label your independent and dependent variables.*

THE BIOLOGY OF CIRCUIT FORMATION

How do proteins control circuit formation?

Proteins encoded by genes allow cells to communicate and respond to one another and their environment. In wild-type flies, neurons express the Roundabout (Robo) receptor on the surface of their axons. The Robo receptor is a protein that allows neurons to detect and respond to the Slit protein that is present at the midline. This Slit->Robo signal prevents the axons of these neurons from re-crossing the midline.

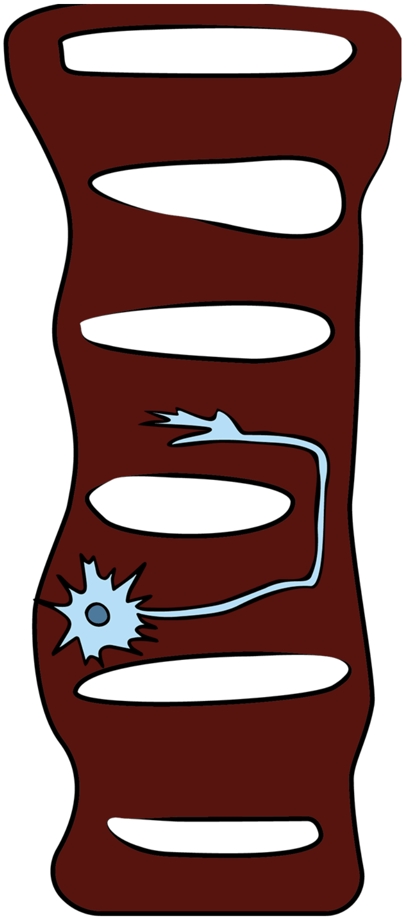

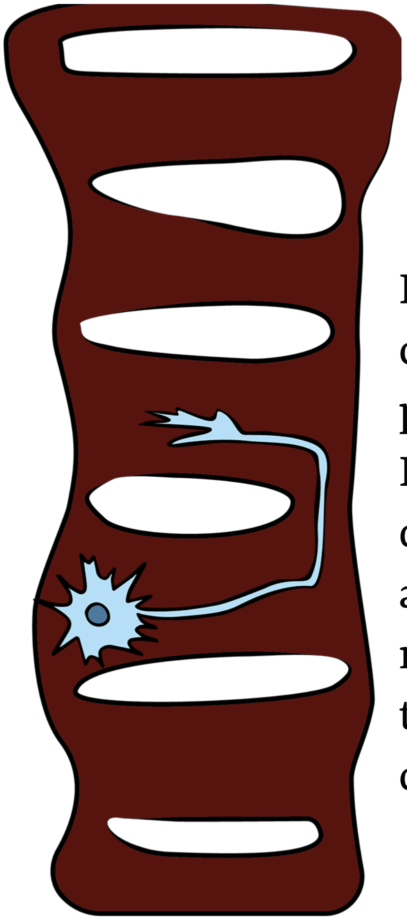

In flies with mutations in the robo gene, changes in the DNA sequence prevent the normal production of the Robo protein. Without Robo, the axons of the neurons can no longer detect, or "see", the Slit signal, and their axons abnormally re-cross the midline. This abnormal midline crossing results in a build-up of axons at the midline, and the crossing bundles of the nerve cord appear thicker and clumped together.

WHAT IS HAPPENING IN THE VIDEO?

In the video, the protein Slit is added to a neuron grown in a dish. Observe the neuron's movements before and after Slit is added, then answer the following questions.

How does the movement of the neuron change once Slit is added?

Would you describe this reaction as "positive" or "negative"? Why?

How would you expect a neuron with no Robo to respond to Slit?

In the video, a scientist added Slit to a neuron growing in a dish. This is called an ***in vitro*** *experiment. When a neuron in growing inside the body, or* ***in vivo****, where does Slit come from?*

LEVELS OF ORGANIZATION

Connecting molecules to behavior

POPULATION

a group of organisms of one type (like flies in a cage)

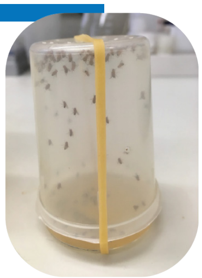

ORGANISM

an individual living thing (like a fly); the basic unit of a species

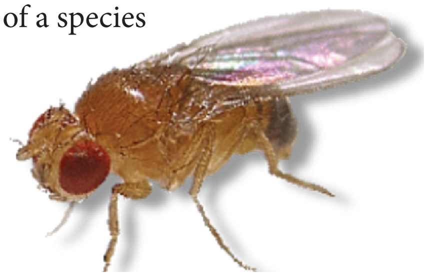

GROUP OF CELLS

Tissues, organs, and organ systems (like the nerve cord pictured here).

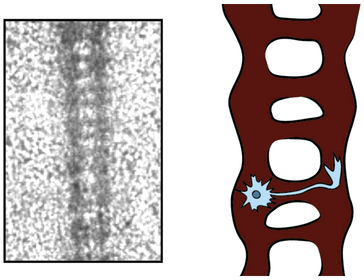

CELLS

The basic unit of life. Neurons (like the one pictured here)are specialized cells.

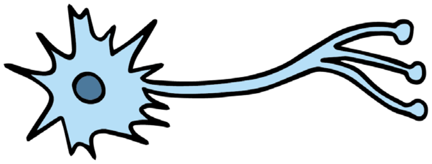

MOLECULES

Groups of atoms. Molecules are the smallest unit of most chemical compounds.

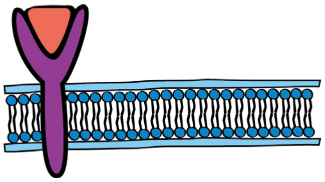

Which level(s) can or did you…

…see with the naked eye?	*…only see with a microscope?*
______________________	______________________
______________________	______________________
____________________	____________________

…not even see with a typical microscope?

____

____

____

…observe in your experiment?

____

____

____

Which level…

…would coordination of limb movements be?

____

____

____

…involves many neurons interacting to create a network to send and receive siganls in an organism?

____

____

NOTES

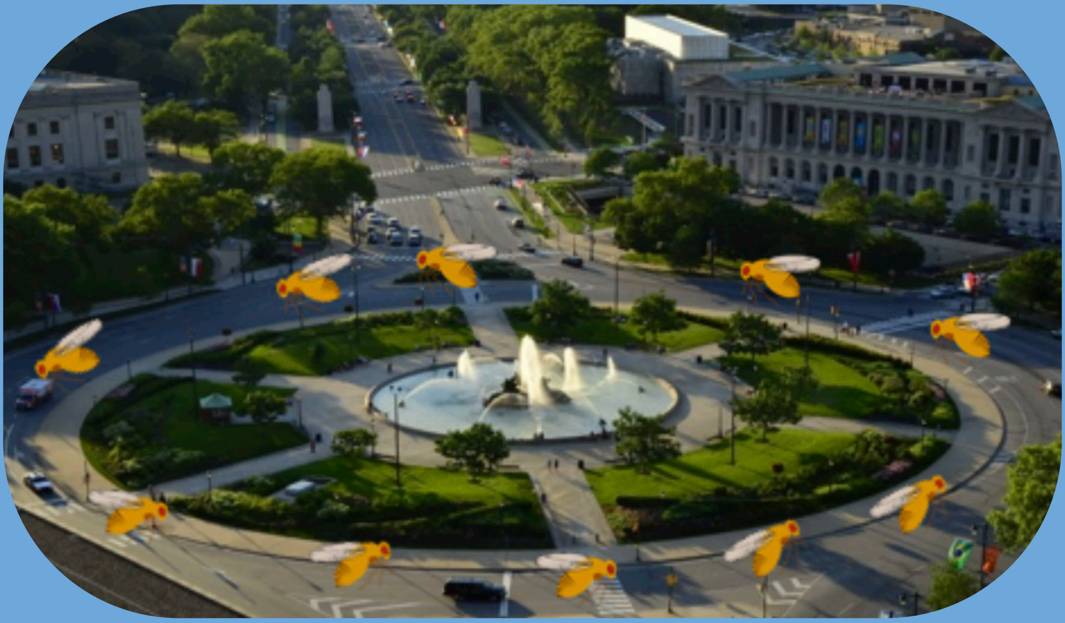

Supplement: Extended Data 4 — Roundabout We Go! student workbook. Download Extended Data 4, ZIP file. [file enu-eN-NWR-0263-22-s03.zip › Supplemental 3 - Roundabout Student Workbook.pdf]
