# Supplementary material for: DrosoPHILA: A Partnership between Scientists and Teachers That Begins in the Lab and Continues into City Schools
Source: eNeuro. 2023 Feb 13;10(2):ENEURO.0263-22.2022. doi: 10.1523/ENEURO.0263-22.2022 (PMC9927510; doi:10.1523/ENEURO.0263-22.2022)
Supplement: Extended Data Table 3-1 — A list of all premodule and postmodule survey questions used to analyze module effectiveness. Download Table 3-1, DOCX file. [file enu-eN-NWR-0263-22-s05.docx]

**Extended Data Table 3-1. Multiple-choice knowledge questions presented in pre- and post-module Google forms.** To assess content knowledge before and after each module, students completed a Google form. The percentage of students who answered each question correctly are presented by topic in Figure 3.

| **Topic** | **Module^a^** | **Question** | **Possible responses^b^** |
| --- | --- | --- | --- |
| **Importance of the nervous system** | Flies on Ice | Why is it important to understand how the nervous system works? | *Diseases of the nervous system are treatable once we understand how they are caused* |
|  |  |  | Our nervous system is not important for processing of our senses |
|  |  |  | The nervous system is the only organ system in humans essential to life |
|  |  |  | Dysfunctions of the nervous system do not cause disease |
|  | Roundabout We Go! | Why is it important to understand how the nervous system works? | Many diseases result from nervous system dysfunction |
|  |  |  | Our senses are processed through the nervous system |
|  |  |  | We can develop therapies for neurological deficits |
|  |  |  | *All of the above* |
| **How neurons communicate** | Flies on Ice | The nervous system is composed of many neurons, which are specialized cells that can communicate through ______ signals | *Electric and chemical* |
|  |  |  | Light |
|  |  |  | Radioactive |
|  |  |  | Magnetic |

| **Fruit flies as model organisms** | Flies on Ice | Why do scientists use fruit flies as a model organism for research? | Fruit flies have a long life cycle like humans |
| --- | --- | --- | --- |
|  |  |  | *Fruit flies are inexpensive and easy to maintain in large numbers* |
|  |  |  | Humans and fruit flies have the exact same number of chromosomes |
|  |  |  | Humans and fruit flies do not share similar genes and diseases |
| **Identifying variables** | Roundabout We Go! | A group of scientists are testing how a specific DNA mutation might affect an organism’s crawling phenotype. What is the independent variable in this experiment? | The crawling patterns of the organisms. |
|  |  |  | The temperature of the room. |
|  |  |  | *The genotypes of the organisms.* |
|  |  |  | The time the organism spends crawling. |
|  | Flies on Ice (pre-module survey) | The independent variable is an experiment is defined as: | The time it takes to design an experiment |
|  |  |  | The variable whose variation does depend on another |
|  |  |  | *The variable whose variation does not depend on another* |
|  |  |  | The variable whose variation is held constant |
|  | Flies on Ice (post-module survey) | You just conducted an experiment to see if the amount of time flies spend on ice affects their recovery response. The ______ is the independent variable in this experiment. | *Amount of time flies spend on ice* |
|  |  |  | Amount of time the flies stay immobilized |
|  |  |  | Number of flies that die |
|  |  |  | Number of flies you put on ice |

| **Experimental Design** | Flies on Ice | Which of the following is a correct statement about designing an experiment? | All experiments require expensive laboratory equipment |
| --- | --- | --- | --- |
|  |  |  | Data from one trial is enough to draw a conclusion |
|  |  |  | Experiments can only be designed by someone with a Ph.D. |
|  |  |  | *Identifying your variables before starting an experiment is important* |
| **Interpreting graphs** | Flies on Ice | Describe the relationship between the time a fly spends on ice and its recovery time as depicted by the graph^c^. | *As time on ice increases, recovery time continues to increase constantly* |
|  |  |  | As time on ice increases, recovery time decreases |
|  |  |  | *As time on ice increases, recovery time increases constantly at first but then remains constant* |
|  |  |  | Time spent on ice and recovery time are not correlated |
| **Mendelian inheritance** | Roundabout We Go! | If two heterozygous flies with the same recessive mutation mate, what percentage of the offspring will display a mutant phenotype? | 10% |
|  |  |  | *25%* |
|  |  |  | 50% |
|  |  |  | 100% |
| **Molecular biology** | Roundabout We Go! | Which correctly describes the events of protein production in the cell? | The gene is translated into mRNA, then transcribed into protein. Finally, the protein carries out its function in the cell |
|  |  |  | The gene for a signal receptor protein gets translated into protein, then transcribed into mRNA. Finally, the protein carries out its function in the cell. |
|  |  |  | *The gene is transcribed into mRNA, and then translated into protein. Finally, the protein carries out its function in the cell.* |

^a^Knowledge questions asked on surveys varied depending on the module instructed, but sometimes shared knowledge areas.

^b^Correct responses are indicated in italics

^c^The graph that students are asked to interpret differs in the pre- and post-module survey. The first graph depicts a linear relationship, and the second graph depicts expected results for Flies on Ice (i.e., a relationship that is initially linear, but begins to plateau as the X axis values increase).
